# Bibliometric Analysis and Systematic Review of Global Coronavirus Research Trends Before COVID-19: Prospects and Implications for COVID-19 Research

**DOI:** 10.3389/fmed.2021.729138

**Published:** 2021-11-16

**Authors:** Peijing Yan, Meixuan Li, Jing Li, Zhenxing Lu, Xu Hui, Yuping Bai, Yangqin Xun, Yongfeng Lao, Shizhong Wang, Kehu Yang

**Affiliations:** ^1^Department of Epidemiology and Health Statistics, West China School of Public Health and West China Fourth Hospital, Sichuan University, Chengdu, China; ^2^Evidence-Based Medicine Center, School of Basic Medical Sciences, Lanzhou University, Lanzhou, China; ^3^Evidence Based Social Science Research Center, School of Public Health, Lanzhou University, Lanzhou, China; ^4^Institute of Medical Research, Northwestern Polytechnical University, Xi'an, China; ^5^School of Basic Medicine, Gansu University of Chinese Medicine, Lanzhou, China; ^6^Department of Scientific Research, Gansu Provincial Hospital, Lanzhou, China; ^7^Department of Pathology, 940th Hospital of Joint Logistics Support Force of Chinese People's Liberation Army, Lanzhou, China; ^8^Key Laboratory of Evidence Based Medicine and Knowledge Translation of Gansu Province, Lanzhou, China; ^9^WHO Collaborating Centre for Guideline Implementation and Knowledge Translation, Lanzhou University, Lanzhou, China; ^10^Department of Urology, Lanzhou University Second Hospital, Lanzhou, China; ^11^Department of Orthopedics, Wuwei People's Hospital, Wuwei, China

**Keywords:** Coronaviruses, COVID-19, bibliometric analysis, systematic review, contribution, research topics

## Abstract

Coronaviruses (CoV) cause respiratory and intestinal infections. We conducted this bibliometric analysis and systematical review to explore the CoV-related research trends from before COVID-19. We systematically searched the Ovid MEDLINE, Ovid Embase, and Web of Science (WOS) databases for published bibliometric analyses of CoV from database inception to January 24, 2021. The WOS Collection was searched from inception to January 31, 2020, to acquire the CoV-related publications before COVID-19. One-Way ANOVA and Bonferroni multiple-comparison tests were used to compare differences. Visualization mapping and keyword cluster graphs were made to illustrate the research topics and hotpots. We included 14,141 CoV-related publications for the bibliometric analysis and 16 (12 articles) CoV-related bibliometric analyses for the systematic review. Both the systematic review and bibliometric analysis showed (1) the number of publications showed two steep upward trajectories in 2003–2004 and in 2012–2014; (2) the research hotpots mainly focused on the mechanism, pathology, epidemiology, clinical diagnosis, and treatment of the coronavirus in MERS-CoV and SARS-Cov; (3) the USA, and China; the University of Hong Kong; and Yuen KY, came from the University of Hong Kong contributed most; (4) the *Journal of Virology* had the largest number of CoV related studies. More studies should focus on prevention, diagnosis, and treatment in the future.

## Introduction

Coronaviruses (CoV) are a large family of positive-sense single-stranded RNA viruses that cause illnesses ranging from the common cold to more severe diseases (1, 2). Some CoV are zoonotic and can cause respiratory and intestinal infections in animals and humans (3), and have even resulted in lethal endemics, such as Middle East Respiratory Syndrome Coronaviruses (MERS-CoV), Severe Acute Respiratory Syndrome Coronaviruses (SARS-CoV), and Coronavirus Disease 2019 (COVID-19) (4).

With the outbreak and epidemic of CoV-related diseases, an increasing number of studies discussed the epidemic characteristics, diagnosis, infection mechanisms, and prevention of CoV (4–8). The appearance of COVID-19 was accelerating such research, which was certainly unique in the history of science and led to an explosion of research output. This output includes many meaningful approaches, but some appear to be excessive and not scientifically sound (9, 10). Against this background, it is very necessary to think about these compelling questions: Can we learn from previous research patterns regarding CoV? What influence do they have on future research? How can we use past efforts, their intensification, and the influences of research on CoV positively to better understand the needs for sustainable and appropriate research? (9). Therefore, it is very important to know about the global research on CoV in the time before COVID-19.

Systematically summarizing and analyzing the research of the CoV is helpful to understand the current state of research and provide references for future research. Bibliometric analysis is a statistical tool that is used to quantitatively and qualitatively measure and evaluate scientific publications (11–13). It consists of a review of the literature, and indicates the number, evaluation, and main trends of publications concerning a specific subject (14, 15).

To the best of our knowledge, there have been two bibliometric studies on CoV-related research in English before the COVID-19 pandemic (16, 17). One study published in 2016 assessed the characteristics of publications only focused on the MERS-CoV (18). Another study (19) analyzed the global research trends of the World Health Organization's top eight emerging infections including Ebola, Marburg, MERS, Severe Acute Respiratory Syndrome (SARS), and so on, but publications related to CoV were not systematically analyzed. A letter to the editor had simply investigated the publication characteristics of SARS-CoV, MERS-CoV, and COVID-19, but it only analyzed the number of publications and countries, which might not be enough to provide a reference for future research (20). In addition, several studies on coronavirus research trends were published in the time before the COVID-19 pandemic. However, some of the research focused on the specific periods, such as 2003 to March 2020. Therefore, we did a bibliometric analysis of all the publications before COVID-19. Additionally, these studies were based on various timespan and databases, and the findings did not well agree. We did this systematic review to summarize the findings of all the current bibliometric analyses in this topic to provide references for researchers focused on the emerging human CoV, and to provide ideas for finding effective control measures, drugs, and vaccines.

## Methods

This is a bibliometric analysis and systematic review, and the data we used were extracted from publications. Therefore, this study has no discernible ethical issues.

### Data Source and Search Strategy

We searched PubMed, Cochrane Library, and Embase databases using the Medical Subject Headings (MeSH) to acquire the CoV-related terms. For the bibliometric analysis, we searched publications using these terms in the Web of Science (WOS) Core Collection from its inception to January 31, 2020. In terms of the systematic review, we systematically searched the Ovid MEDLINE, Ovid Embase, and WOS databases using terms relating to CoV and bibliometric analysis, for published bibliometric analysis from database inception to January 24, 2021. The detailed search strategy is displayed in Appendix (Appendix, Supplementary Tables 1, 2). No limitation was used. As the metrics are changing over time, all the searches and data exports were completed on the same day to avoid the possible bias caused by frequent updates of the databases.

### Eligibility Criteria for Systematic Review

This systematic review included the bibliometric analyses of global CoV research trends. We excluded the bibliometric analyses without any indicators of publication and citation, journal, country or territory, affiliation and international cooperation, author, or subject/research topic. We also excluded conference abstracts, editorials, reviews, meta-analyses, and case reports or case series, as well as non-English and non-Chinese language publications and publications reporting duplicate data.

### Data Collection and Cleaning

In terms of bibliometric analysis, we obtained (1) the characteristics of all the retrieved publications; (2) the 2019 journal impact factor (JIF) (21), 5 year JIF (21), and publication counts of the journals; (3) publication count per year, h-index, various citation values [average citations per item (ACPI), sum of times cited (STC) and No. citations of most-cited item (NCMCI)] and top-5 most-publications research areas (top-5 research areas) of the top-10 most-publications countries (top-10 countries); and (4) institutes, h-index, various citation values, and top-5 research areas of the top-10 most-publications authors (top-10 authors). All documents were downloaded in tab separator format.

We standardized the keywords with the same meaning but in different styles. For example, “coronavirus” was replaced by “coronavirus (cov)”, “middle east respiratory syndrome coronavirus” was replaced by “MERS”, etc.

As for the systematic review, one researcher (Y-PB) extracted the information from the included studies using a pre-piloted, standardized extraction table, and the other researcher (P-JY) checked the extraction. Any discrepancies between the reviewers were resolved by discussion. We extracted the following information: (1) study characteristics (first author, publication year, country, journal); (2) search strategies, and (3) indicators or findings on publication and citation, journal, country or territory, affiliation and international cooperation, author, subject/research topics, and keyword co-occurrence cluster.

Since there is no validated quality assessment tool that can be applied to bibliometric analyses, we did not assess the risk of bias or the methodological quality for the included bibliometric analyses.

### Statistical Analysis

The data were entered into a spreadsheet program (Microsoft Excel 2016, Microsoft, Washington, USA). The statistical analyses and preparation of the figures were performed using Stata, version 15 (Stata Corp, College Station, TX, USA). For all statistical tests, a two-tailed α level of 0.05 was used.

We used VOSviewer 1.6.1 (Centre for Science and Technology Studies, Leiden University, Leiden, The Netherlands) to analyze the publication characteristics (22, 23). Keywords co-occurrence can effectively reflect the research hotspots in the discipline fields, providing auxiliary support for scientific research (24). VOSviewer was also used for visualization mapping to present co-authorship and co-occurrence networks (25) and generate keywords clustering graph to present the research topic.

## Results

### Basic Characteristics of CoV–Related Publications

A total of 14,141 publications were retrieved, of which around 77.27% were published as original articles, 8.36 % as reviews, 3.91% as proceedings papers, 3.13% as meeting abstracts, with the remaining being book chapters, etc., (Appendix, Supplementary Figure 1). For the book chapters, the Advances in Experimental Medicine and Biology (273), Advances in Virus Research (26), and Current Topics in Microbiology and Immunology (27) were the top-3 most-publications, others were less than 10 records.

Among these publications, 53.35% (7,544) records did not contain data in the funding agencies; 97.24 % (13,750) were published in English, 0.79 % (111) were in French, 0.75 % (106) were in German, and the remaining were in Spanish, Chinese, and 14 other languages.

### The Annual Trends of CoV–Related Publications

**Figure 1:**
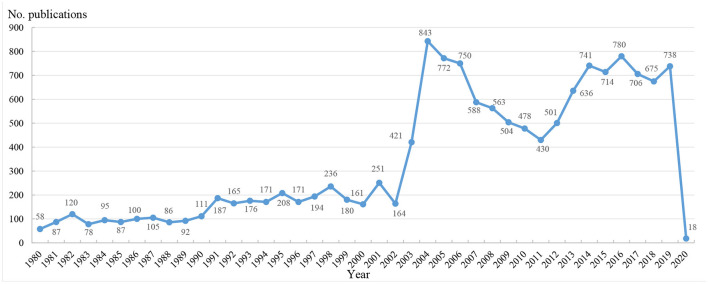
Annual trends of CoV-related publications.

Figure 1 plots the annual trends of CoV-related publications. Since the first literature was published in 1980, CoV-related research had a very slow increase in the following 20 years. The number of publications grew very sharply in 2003, hit a peak in 2004 (843), and then declined gradually until another sudden increase in 2012 (Figure 1).

### Journals of CoV–Related Publications

**Figure 2:**
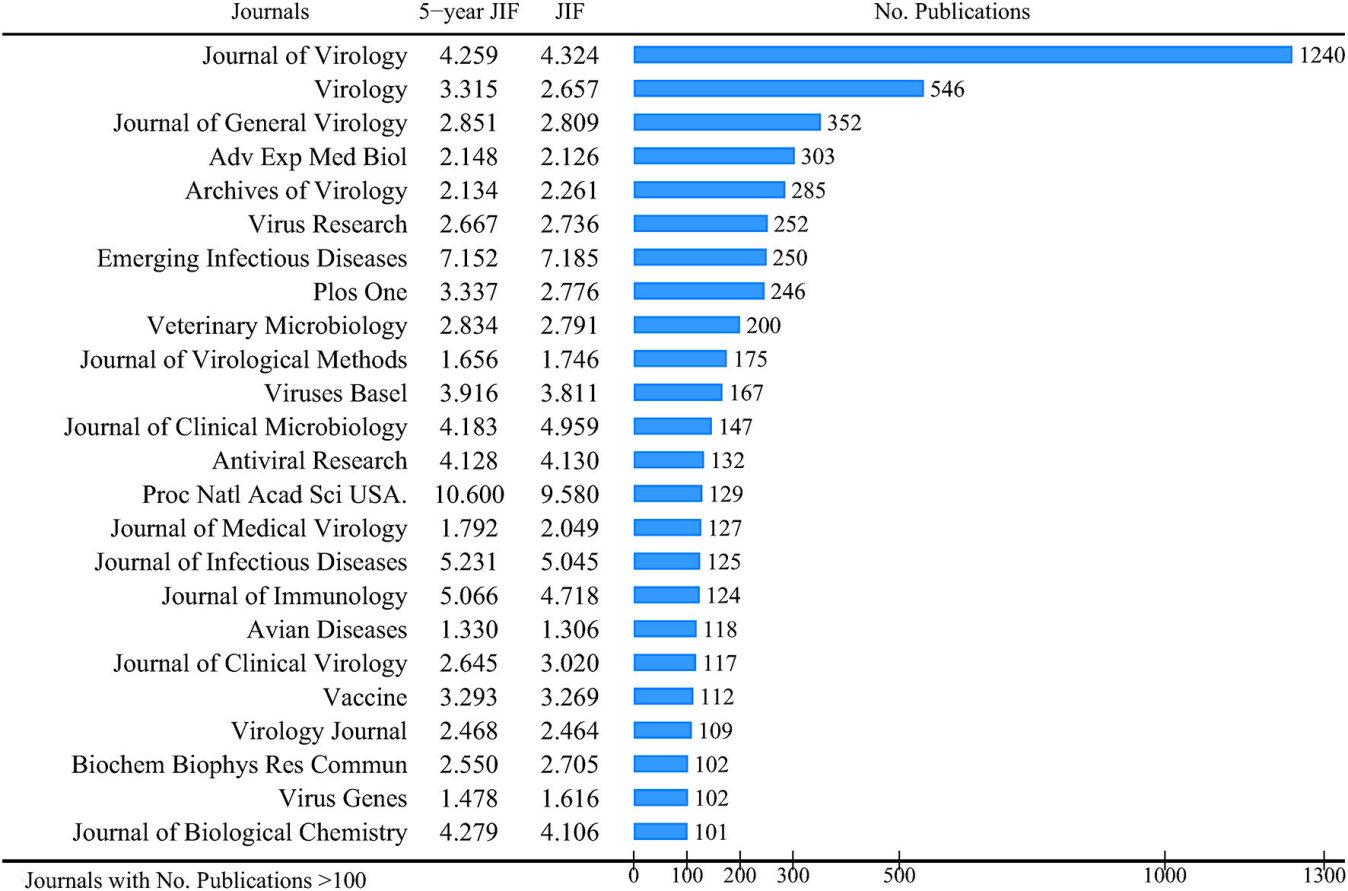
Journals with more than 100 CoV-related publications.

The CoV-related publications were published in 500 journals. The 24 journals with more than 100 publications were listed. The journal with the most publications was the Journal of Virology (1,240), followed by Virology (546) and the Journal of General Virology (352). The 2019 JIF ranged from 1.306 (Avian Disease) to 9.580 (Proceedings of The National Academy of Science of The United States of America), and the 5 year JIF ranged from 1.330 to 10.600 (Figure 2).

### Countries and Regions of CoV–Related Publications

**Figure 3:**
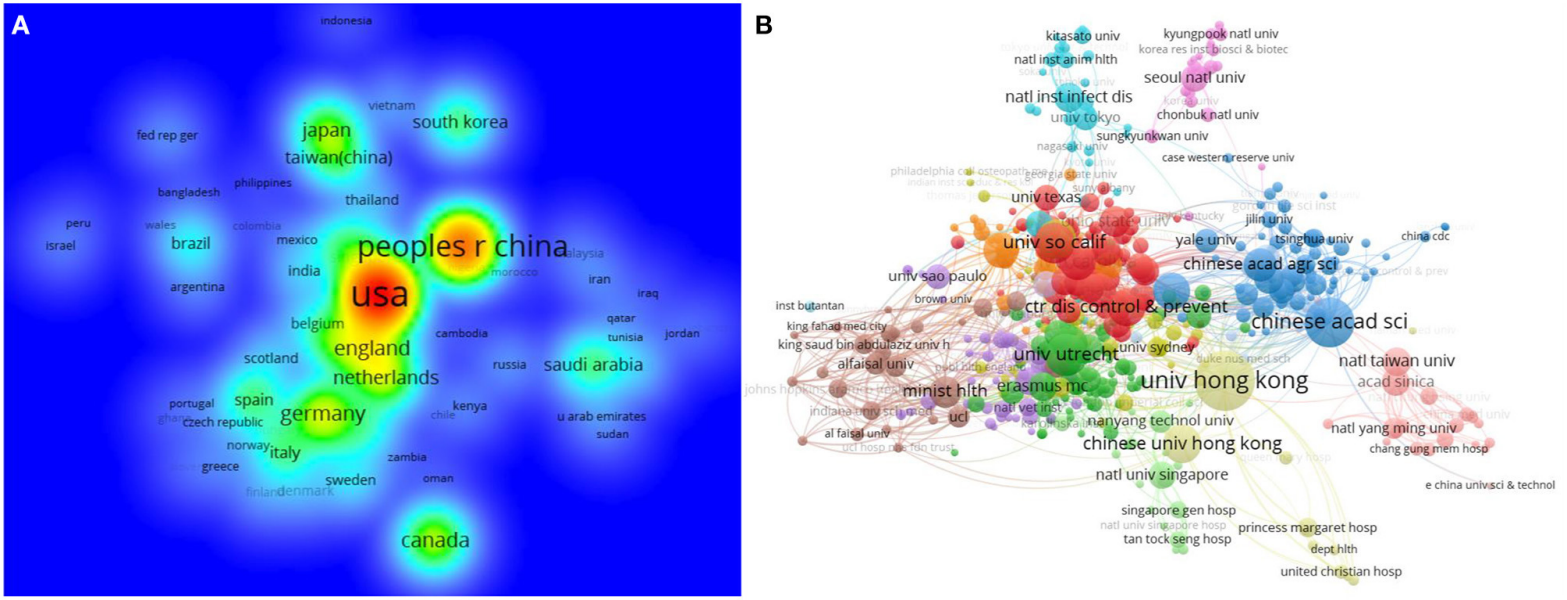
Country **(A)** and institution **(B)** co-authorship network of CoV-related publications.

A total of 134 countries published CoV-related studies. Around 32.49% of those publications were published in North America, 31.49% in Europe, 30.78% in Asia, and the remaining in Oceania, South America, and other regions (Appendix, Supplementary Figure 2). The cooperation network analysis included 88 countries, which with a frequency ≥ 5 times. The density map showed that the top-10 countries were the United States of America (USA) with 5,142, followed by China (2,754), Germany (961), Canada (887), England (880), Netherlands (788), Japan (710), France (647), South Kores (438), and Taiwan (China) (422) (Figure 3A). Among the top-10 countries, 1/5 were from North America, 2/5 from Asia, and the rest from Europe (Table 1). The CoV-related publication count of the top-10 countries over the 41 years is listed in Appendix (Appendix, Supplementary Table 3).

**Table 1:** Characteristics of top-10 countries (*N* = 14,141).

**ID**	**Country**	***N*** **(%)**	**Region**	**NCMCI**	**Top-5 Research areas**
1	USA	5,142 (36.36)	North America	1,823	Virology; Immunology; Veterinary Sciences; Microbiology; Biochemistry Molecular Biology
2	China	2,754 (19.48)	Asia	1,823	Virology; Biochemistry Molecular Biology; Immunology; Infectious Diseases; Microbiology
3	Germany	961 (6.80)	Europe	1,732	Virology; Immunology; Biochemistry Molecular Biology; Veterinary Sciences; Infectious Diseases
4	Canada	887 (6.27)	North America	1,273	Virology; Immunology; Veterinary Sciences; Biochemistry Molecular Biology; Infectious Diseases
5	England	880 (6.22)	Europe	1,328	Virology; Veterinary Sciences; Infectious Diseases; Biochemistry; Molecular Biology Immunology
6	Netherlands	788 (5.57)	Europe	1,732	Virology; Microbiology; Infectious Diseases; Immunology; Biochemistry Molecular Biology
7	Japan	710 (5.02)	Asia	794	Virology; Veterinary Sciences; Immunology; Microbiology; Biochemistry Molecular Biology
8	France	647 (4.58)	Europe	1,732	Virology; Infectious Diseases; Veterinary Sciences; Immunology; Microbiology
9	South Korea	438 (3.10)	Asia	320	Virology; Infectious Diseases; Veterinary Sciences; Microbiology; Immunology
10	Taiwan (China)	422 (2.98)	Asia	1,823	Biochemistry Molecular Biology; Virology; Infectious Diseases; Pharmacology Pharmacy; Immunology

*NCMCI, No. citations of most-cited item*.

Among these countries, a total of 6,753 institutions were involved in CoV-related publications. A network of 530 institutions with a frequency ≥ 10 was formed. The University of Hong Kong (China), the Chinese Academy of Sciences (China), Utrecht University (Netherlands), the University of Southern California (USA), and the University of Pennsylvania (USA) were at the center of the cooperation network and formed close cooperative relationships with other institutions (Figure 3B).

In terms of STC, h-index, and NCMCI of the top-10 countries, the USA was the most-contributed country with the highest h-index (156), STC (185,165), and NCMCI (1,823), followed by the Netherlands (107) and China (105) in h-index, China (73,101) and the Netherlands (47,486) in STC, and China (1,823) and Taiwan (1,823) in NCMCI (Table 1).

The CoV-related publications of the top-10 countries mainly focused on the following research areas: virology, veterinary sciences, infectious diseases, immunology, biochemistry molecular biology, microbiology, and pharmacology (Table 1, Appendix, Supplementary Figure 3). The most-contributed research area of the top-10 countries was virology, except for Taiwan, which focused on biochemistry molecular biology. The Netherlands contributed more to the virology area than any of the other nine countries (Appendix, Supplementary Figure 3).

### Authors of CoV–Related Publications

**Figure 4:**
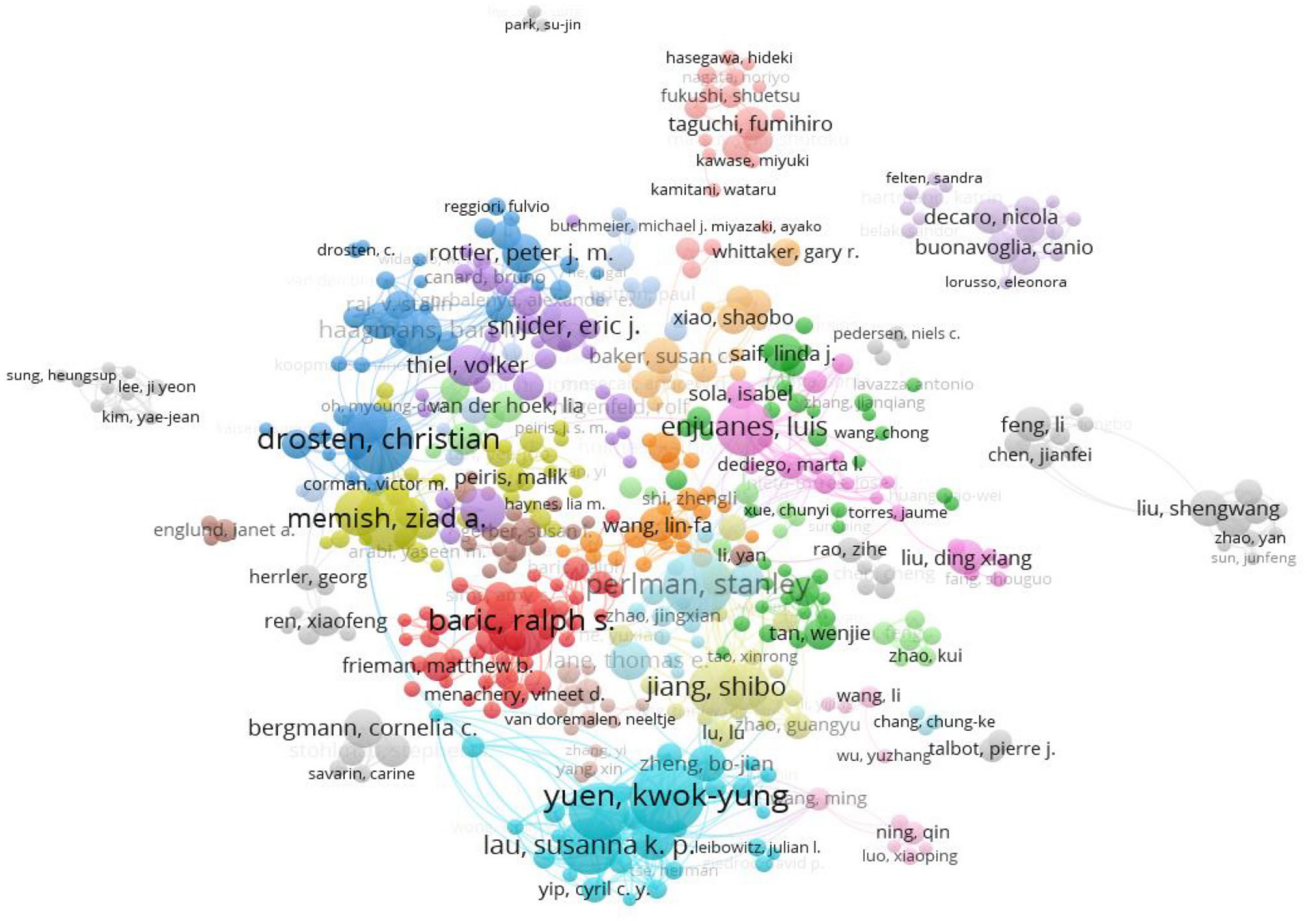
Co-authorship network of CoV-related publications.

A total of 43,476 authors were involved in the CoV-related publications, 402 authors with a frequency ≥ 10 times were included in the collaboration network analysis, and 27 cooperation networks were formed. Yuen KY (China), Baric RS (USA), and Drosten C (Germany) had the highest number of publications and were in the middle of the network diagram, which shows that they formed close cooperative relationships with other authors (Figure 4).

**Figure 5:**
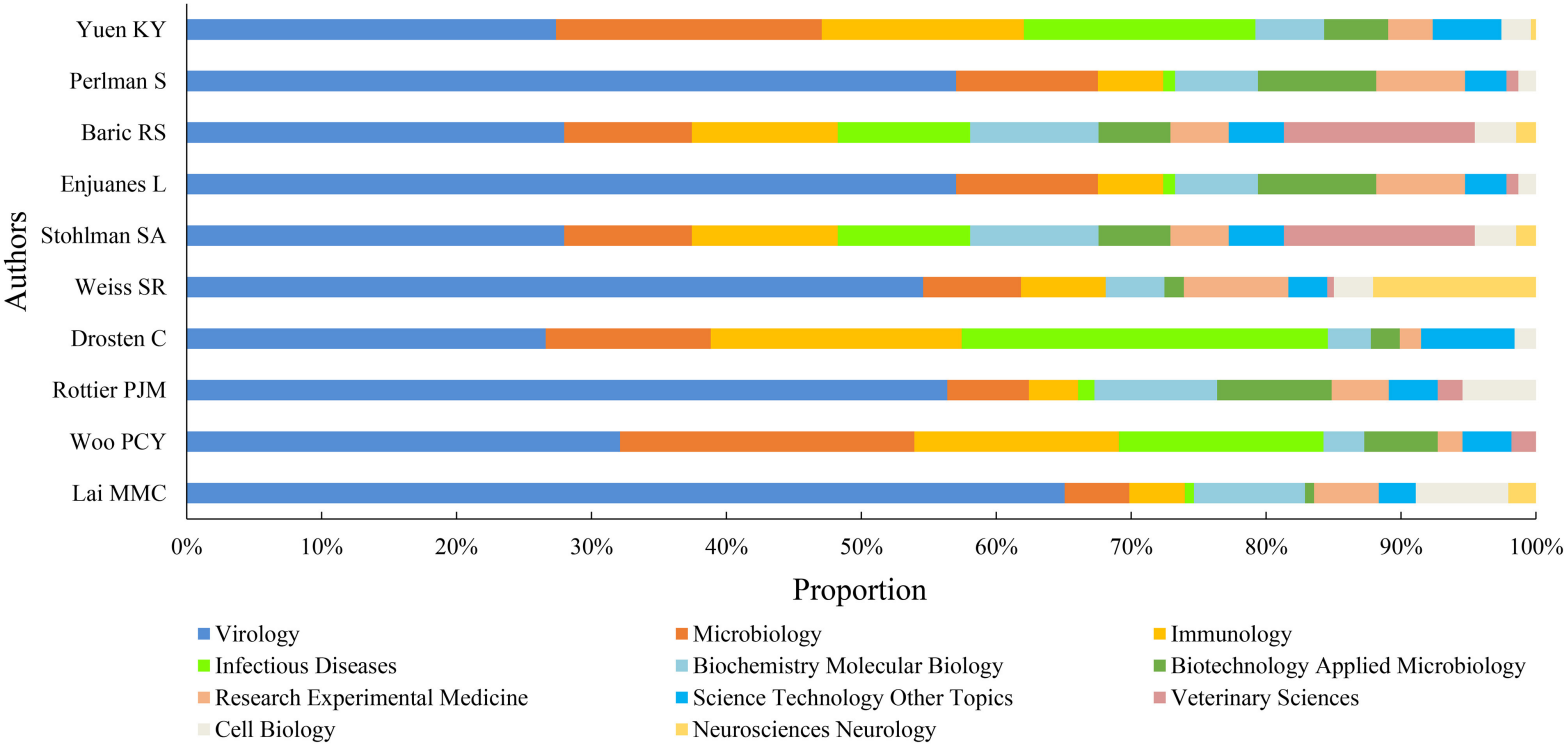
Proportion of top-5 research areas for the top-10 authors.

The top-10 authors with the most CoV-related publications mainly came from the USA (1/2) and China (1/5), and were focused in the University of Southern California and the University of Hong Kong (Table 2). Most of the top-10 authors came from the departments of microbiology (Table 2) and mainly focused on virology, microbiology, infectious diseases, immunology, and seven other research areas (Figure 5, Table 2). The most-contributed research area of the top-10 authors was virology, except Drosten C who focused on infectious diseases (Figure 5).

**Table 2:** Characteristics of the top-10 authors.

**ID**	**Author**	***N*** **(%)**	**Organization**			**H-Index**	**Citation**			**Top-5 Research areas**
			**Country**	**Institute**	**Department**		**ACPI**	**STC**	**NCMCI**	
1	Yuen KY	213 (1.51)	China	University of Hong Kong	Department of Microbiology and Pathology	67	76.10	16,210	1,436	Virology; Microbiology; Infectious Diseases; Immunology; Biochemistry Molecular Biology
2	Perlman S	187 (1.32)	USA	University of Iowa	Department of Microbiology	44	32.13	6,008	321	Virology; Microbiology; Biotechnology Applied Microbiology; Research Experimental Medicine; Biochemistry Molecular Biology
3	Baric RS	170 (1.20)	USA	University of North Carolina	Department of Epidemiology	53	43.61	7,413	321	Virology; Veterinary Sciences; Immunology; Infectious Diseases; Biochemistry Molecular Biology
4	Enjuanes L	162 (1.15)	Spain	Centro Nacional de Biotecnologia	Department of Molecular and Cell Biology	48	40.62	6,580	350	Virology; Microbiology; Biotechnology Applied Microbiology; Research Experimental Medicine; Biochemistry Molecular Biology
5	Stohlman SA	156 (1.10)	USA	University of Southern California	Departments of Microbiology and Neurology	52	48.13	7,508	266	Virology; Veterinary Sciences; Immunology; Infectious Diseases; Biochemistry Molecular Biology
6	Weiss SR	156 (1.10)	USA	University of Pennsylvania School of Medicine	Department of Microbiology	44	33.74	5,263	237	Virology; Neurosciences Neurology; Research Experimental Medicine; Microbiology; Immunology
7	Drosten C	144 (1.02)	Germany	National Reference Center for Tropical Infectious Diseases	Bernhard Nocht Institute for Tropical Medicine	48	76.30	10,987	1,732	Infectious Diseases; Virology; Immunology; Microbiology; Science Technology Other Topics
8	Rottier PJM	134 (0.95)	Netherlands	Utrecht University	Department of Infectious Diseases & Immunology	50	56.95	7,631	459	Virology; Biochemistry Molecular Biology; Biotechnology Applied Microbiology; Microbiology; Cell Biology
9	Woo PCY	127 (0.90)	China	University of Hong Kong	Department of Microbiology	47	58.57	7,438	646	Virology; Microbiology; Immunology; Infectious Diseases; Biotechnology Applied Microbiology
10	Lai MMC	123 (0.87)	USA	University of Southern California	Department of Molecular Microbiology and Immunology	56	67.42	8,293	545	Virology; Biochemistry Molecular Biology; Cell Biology; Microbiology; Research Experimental Medicine

*ACPI, Average citations per item; STC, Sum of times cited; NCMCI, No. citations of most-cited item*.

Yuen KY had the highest number of publications, h-index, and STC, followed by Perlman S and Baric RS in number of publications, Lai MMC and Baric RS in h-index, and Drosten C and Lai MMC in STC. Drosten C had the highest NCMCI and ACPI, followed by Yuen KY and Woo PCY in NCMCI and Yuen KY and Lai MMC in ACPI (Table 2).

### Research Topics of CoV–Related Publications

**Figure 6:**
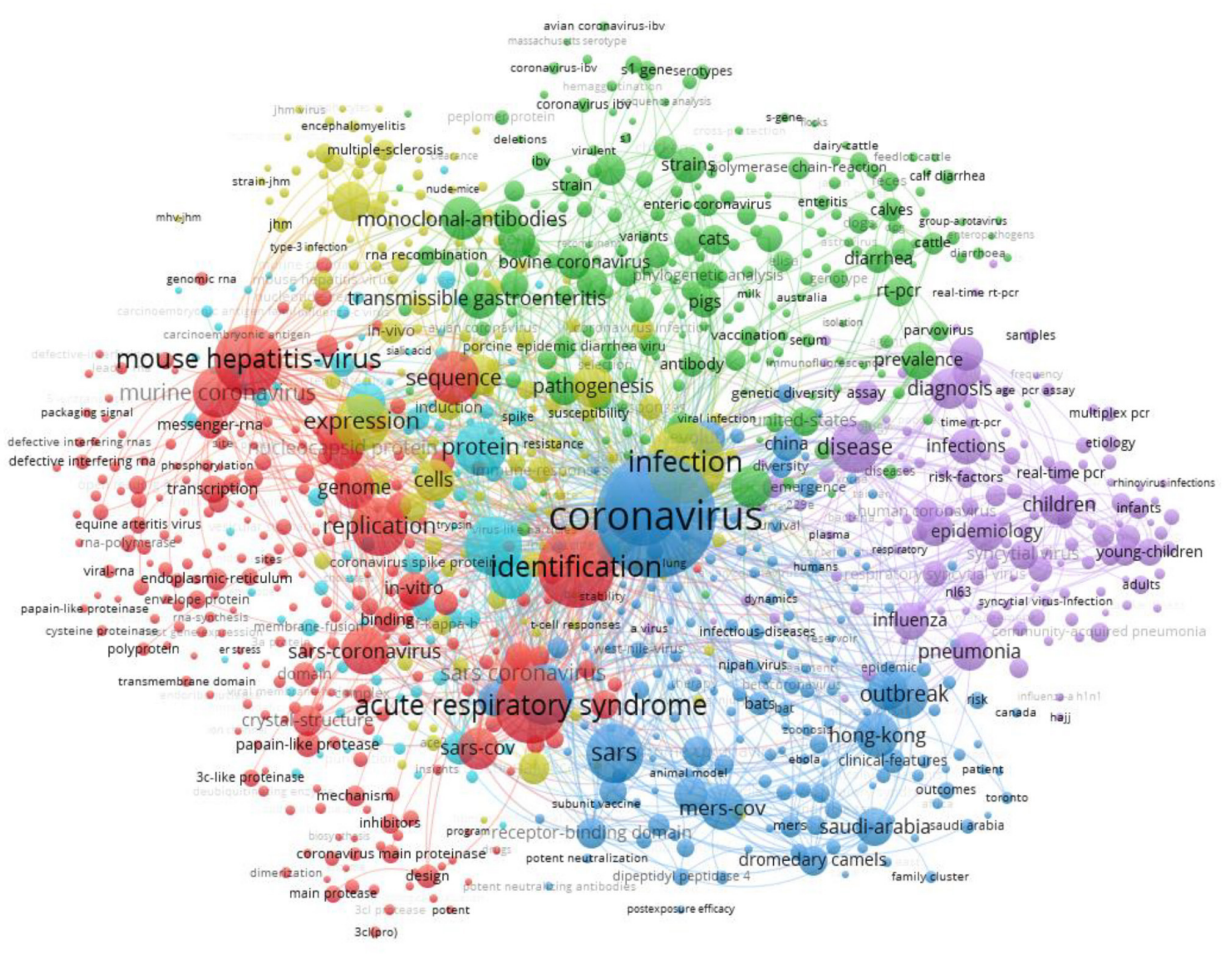
Keyword co-occurrence network in CoV-related publications.

A total of 23,732 keywords were included in the 14,141 publications, and 973 keywords with occurrence frequency ≥ 20 were clustered. In the cluster figure, one type of color represents one cluster, and a total of five main clusters were formed, indicating that the current CoV-related research concentrated on the following five topics: Topic 1 (red area, 239 items): the detection and identification of SARS-CoV by collecting nucleic acid and protein of virus *in vitro*; Topic 2 (green area, 211 items): research on the natural history, transmission, and diagnosis of CoV; Topic 3 (blue area, 166 items): research on SARS-CoV outbreaks in China, and the MERS-CoV outbreak in Saudi Arabia; Topic 4 (yellow area, 138 items): research on the mechanisms of viral infection and expression in *in vitro* cells and lab mice; and Topic 5 (purple area, 133 items): research on pneumonia caused by human infection with CoV and the spread, prevalence, and burden of various diseases caused by infection with other viruses such as avian influenza (Figure 6).

The density map of 973 keywords is presented in the Appendix (Appendix, Supplementary Figure 4). “CoV (3,116 items),” “infection (1,413 items),” “identification” (1,393 items), etc. had the highest frequency in the red area, followed by “diagnosis (395 items),” and the “therapy (69 items)” in the yellow area and “treatment (35 items)” in the green area (Appendix, Supplementary Figure 4).

### High-Frequent Citation Articles

Most of the top-10 most-cited publications came from the USA and were in high impact-factor journals such as New England Journal of Medicine, British Medical Journal, and Science. The most frequently cited article (1,823 citations) was published by Ksiazek et al. (28), followed by Drosten et al. (29) (1,732 citations) (Table 3).

**Table 3:** The top-10 most-cited publications.

**ID**	**Title**	**Publication year**	**First author**	**Country**	**Institution**	**STC**	**Journal**
1	A novel coronavirus associated with severe acute respiratory syndrome	2003	Ksiazek TG	USA	CDC, the Special Pathogens Branch	1,823	NEJM
2	Identification of a novel coronavirus in patients with severe acute respiratory syndrome	2003	Drosten C	Germany	The Bernhard Nocht Institute for Tropical Medicine	1,732	NEJM
3	Characterization of a novel coronavirus associated with severe acute respiratory syndrome	2003	Rota PA	USA	National Center for Infectious Diseases, CDC	1,487	Science
4	Coronavirus as a possible cause of severe acute respiratory syndrome	2003	Peiris JSM	China	University of Hong Kong, Queen Mary Hospital	1,436	Lancet
5	Community study of role of viral infections in exacerbations of asthma in 9–11 years old children	1995	Johnston SL	British	University Medicine, Southampton General Hospital	1,328	BMJ
6	Isolation of a Novel Coronavirus from a Man with Pneumonia in Saudi Arabia	2012	Zaki AM	Saudi Arabia	The Dr. Soliman Fakeeh Hospital	1,274	NEJM
7	The genome sequence of the SARS-associated coronavirus	2003	Marra MA	British	BCCA, Genome Sciences Centre	1,273	Science
8	Cloning of a human parvovirus by molecular screening of respiratory tract samples	2005	Allander N T	Sweden	Karolinska University Hospital	1,011	PNAS
9	Psychological Stress and Susceptibility to the Common Cold	1991	Cohen S	USA	Carnegie Mellon University	1,004	NEJM
10	Angiotensin-converting enzyme 2 is a functional receptor for the SARS coronavirus	2003	Li WH	USA	Division of Pulmonary Medicine and Ina Sue Perlmutter Laboratory	968	Nature

*STC, Sum of times cited; CDC, Centers for Disease Control and Prevention; NEJM, New England Journal of Medicine; BMJ, British Medical Journal; PNAS, Proceedings of the National Academy of Sciences of the United States of America*.

### Systematic Review of Published Bibliometric Analyses

#### Study Characteristics of Published Bibliometric Analyses

A total of 17 (9, 11, 26, 27, 30–38) CoV-related bibliometric analyses from 13 articles were included, of which one study (27) conducted five bibliometric analyses. All 17 bibliometric analyses were published in 2020 and included between 641 and 15,207 primary studies (Table 4). Two included articles from India (26, 38), one from Spain (34), one from Turkey (27), two from Israel (9, 31), and the remaining 7 reports were from China (Appendix, Supplementary Table 4). All the 13 included articles were published in journals with IF from 0 to 5.993 (Appendix, Supplementary Table 4). Most of the CoV-related bibliometric analyses (88.2%, 15/17) retrieved data from the WOS up to 2020, and the types of original studies included were mainly articles and reviews. A majority of the CoV-related bibliometric analyses indicated that the annual publication count increased due to three notable epidemic events in history.

**Table 4:** The characteristics and top-3 information of published bibliometric analyses.

**References**	**Search date-search deadline**	**Dataset**	**No. publications**	**The Top-3 Journal**	**The Top-3 Countries or territories (n, %)**	**The Top-3 Institutions (n)**	**The Top-3 Authors**
(11)	Database inception to Feb-20	Scopus	15207	1. JVI; 2. EID; 3. Lancet	1. USA (4,225, 27.8%); 2. China (mainland) (2,720, 17.9%); 3. China (Hong Kong) (1,411, 9.3%)	1. University of Hong Kong, China (703); 2. Chinese University of Hong Kong, China (499); 3. Chinese Academy of Sciences, China (407)	1. Yuen KY, University of Hong Kong, China (180); 2. Drosten C, Charité-Universitätsmedizin Berlin, Germany (128); 3. Peiris JSM, University of Hong Kong, China (111)
(33)	Jun-05 to Feb-20	WOSCC	9760	1. JVI; 2. Virology; 3. PLoS One	1. USA (3,452, 35.4%); 2. China (2,402, 24.6%); 3. Germany (642, 6.6%)	1. University of Hong Kong, China (959); 2. Chinese Academy of Sciences, China (469); 3. Chinese University of Hong Kong, China (411)	1. Yuen KY, University of Hong Kong, China (200); 2. Baric RS, University of North Carolina, USA (134); 3. Perlman S, University of Iowa, USA (133)
(9)	Database inception to Mar-20	WOSCC	6905	NR	1. USA (2,293, 33.2%); 2. China (1,707, 24.7%); 3. Germany (505, 7.3%)	1. University Hong Kong, China (398); 2. Chinese University Hong Kong, China (217); 3. CDC, USA (155)	NR
(36)	Jan-03 to Apr-20	WOSCC	11036	1. JVI; 2. EID; 3. Virology	1. USA (3,606, 32.7%); 2. China (3,139, 28.4%); 3. Germany (669, 6.1%)	1. University of Hong Kong (595); Chinese University of Hong Kong (311); 2. CDC (266)	1. Yuen, KY, University of Hong Kong, China (214); 2. Drosten C, University of Bonn, Germany (142); 3. 3. Baric RS., University of North Carolina, USA (131)
(37)	Jan-00 to Mar 20	WOSCC	9105	1. JVI; 2. Virology; 3. PLoS One	1. USA (3,101, 34.3%); 2. China (2,230, 24.7%); 3. Germany (584, 6.5%)	1. University of Hong Kong, China (434); 2. Chinese Academy of Science, China (329); 3. University of California System, USA (246)	NR
(30)	Jan-03 to Feb-20	WOSCC	8433	NR	1. USA (2,791, 33.1%); 2. China (2,231, 26.5%); 3. Germany (564, 6.7%)	1. University of Hong Kong, China (399); 2. Chinese Academy Sciences, China (298); 3. CDC, USA (184)	1. Yuen KY, University of Hong Kong, China (178); 2. Drosten C, University of Bonn, Germany (118); 3. Baric RS, University of North Carolina, USA (114)
(32)	Jan-03 to Feb-20	WOSCC	9294	1. JVI; 2. Virology; 3. Virus Research	1. USA (3,225, 34.7%); 2. China (2,410, 25.9%); 3. Germany (621, 6.7%)	1. University of Hong Kong, China (452); 2. Chinese Academy of Sciences, China (323); 3. CDC, USA (197)	1. Yuen KY, University of Hong Kong, China (TLS=598 times); 2. Chan KH, (TLS=411 times); 3. Woo PCY, University of Hong Kong, China (TLS=382 times)
(27)	Jan-80 to Dec-19	WOSCC	13833	1. JVI; 2. Virology; 3. ADV EXP MED BIOL	1. USA (4,894, 35.4%); 2. China (16.7%); 3. Germany (6.7%)	1. University of Hong Kong, China (534); 2. Chinese Academy of Sciences, China (396); 3. Utrecht University, Netherlands (335)	1. Yuen KY, University of Hong Kong, China (218); 2. Perlman S, University of Iowa, USA (189); 3. Enjuanes L, Autonomous University of Madrid, Spain (176)
(27)	Jan-80 to Dec-19	WOSCC	641	JGV	1. USA (36.7%); 2. Germany (13.4%); 3. UK (12.2%)	University of Würzburg, Germany	Termeulen V
(27)	Jan-80 to Dec-19	WOSCC	1674	JVI	1. USA (44.3%); 2. Germany (9.0%); 3. Canada (8.3%)	University of Southern California, USA (96)	Lai MMC, University of Southern California, USA (70)
(27)	Jan-80 to Dec-19	WOSCC	4810	1. JVI; 2. Virology; 3. ADV EXP MED BIOL	1. USA (1,679, 34.9%); 2. China (1,202, 25.0%); 3. Canada (324, 6.7%)	1. University of Hong Kong, China (284); 2. Chinese Academy of Sciences, China (221); 3. Chinese University of Hong Kong, China (172)	Yuen KY, University of Hong Kong, China (110)
(27)	Jan-80 to Dec-19	WOSCC	6601	NR	1. USA (2,218, 33.6%); 2. China (1,479, 22.4%); 3. Germany (436, 6.6%)	1. University of Hong Kong, China (243); 2. National Institutes of Health, USA (184); 3. Chinese Academy of Sciences, China (170)	Drosten C, Charité – Universitätsmedizin, Germany (113)
(26)	Jan-68 to Mar-20	WOS	6424	1. JVI; 2. JGV; 3. Virology	1. USA (2,345, 36.5%); 2. China (1,067, 16.6%); 3. Germany (480, 7.5%)	1. University of Hong Kong, China (506); 2. University of North Carolina, USA (412); 3. Chinese Academy of Sciences, China (371)	NR
(34)	Jan-70 to Apr-20	WOS	12571	1. JVI; 2. Virology; 3. ADV EXP MED BIOL	1. USA (4,513, 35.9%); 2. China (2,746, 21.8%); 3. UK (962, 7.7%)	1. University of Hong Kong, China (487); 2. Chinese Academy of Sciences, China (373); 3. University of California System, USA (321)	1. Yuen KY, University of Hong Kong, China (201); 2. Perlman S, University of Iowa, USA (169); 3. Baric RS, University of North Carolina (162); Enjuanes L, Autonomous University of Madrid, Spain (162)
(31)	Jan-02 to NR	MAG; PubMed; SJR; Wikidata	NR	NR	NR	NR	NR
(35)	Database inception to Feb-20	WOSCC	1747	NR	1. USA (613, 35.4%); 2. China (582, 33.6%); 3. Germany (122, 7.1%)	1. Chinese Academy of Sciences, China (82); 2. University of Hong Kong, China (74); 3. Chinese University of Hong Kong, China (58)	1. Baric RS, University of North Carolina (21); 2. Yuen KY, University of Hong Kong, China (17); Snijder EJ, Netherlands (17); Kuochen Chou, USA (17); 3. Jiang Shibo, China (16)
(38)	Jan-00 to Dec-19	WOS	10816	1. JVI; 2. Virology; 3. EID	1. USA (3,755, 34.6%); 2. China (2,618, 24.1%); 3. Germany (737, 6.8%)	1. University of Hong Kong, China (511); 2. Chinese Academy of Sciences, China (385); 3. National Institute of Health, USA (270)	1. Yuen, KY, Pamela Youde Nethersole Eastern Hospital, Hong Kong (210); 2. Perlman, S, University of Iowa, USA (148); 3. Drosten, C, Bernhard Nocht Institute for Tropical Medicine, National Reference Center for Tropical Infectious Diseases, Hamburg, Germany (144)

*WOSCC, The Web of Science Core Collection; WOS, Web of Science; JVI, Journal of Virology; JGV, Journal of General Virology; EID, Emerging Infectious Diseases; CDC, Center for Disease Control and Prevention; NR, not reported; MAG, Microsoft Academic Graph; ADV EXP MED BIOL, Advances in Experimental Medicine and Biology; SJR, Scientific Journal Rankings; PNAS, Proceedings of the National Academy of Sciences of the United States of America; TLS, total link strength*.

#### Journals, Countries, Institutions, and Authors of CoV-Related Publications in Published Bibliometric Analysis

Six (11, 26, 33, 36–38) of 17 included bibliometric analyses reported the total number of CoV-related research journals (100–3,443), 11 bibliometric analyses (8 articles) (11, 26, 27, 32–34, 36, 37) listed the top 1–20 journals, and all of them reported that the *Journal of Virology* had the largest number of CoV-related studies (Table 4, Appendix, Supplementary Table 5).

In terms of countries, 6 bibliometric analyses (30, 32, 33, 36–38) reported the total number of CoV-related research by country (78–219), and nearly all of them listed the top 20 most-publications countries countries. In 16 of the 17 bibliometric analyses (9, 11, 26, 27, 30, 32–38) it was indicated that the USA had the largest number of CoV-related publications, followed by China, and their cooperative network diagram showed the most frequent cooperation occurred in the USA and China (Table 4, Appendix, Supplementary Table 5).

As for institutions, 4 bibliometric analyses (30, 33, 35, 38) reported the total number of CoV-related research institutions (147, 242, 333, 6,306 respectively), and 12 bibliometric analyses (9, 11, 26, 27, 30, 32–34, 36–38) indicated that the University of Hong Kong had the largest number of CoV-related publications. Only 5 bibliometric analyses (30, 32, 33, 35, 36) analyzed cooperation among institutions, and their conclusions were inconsistent (Table 4, Appendix, Supplementary Table 5).

Regarding authors, 4 bibliometric analyses (30, 32, 33, 35) reported the total number of CoV-related research authors (121-29515), 12 bibliometric analyses listed top-20 authors (11, 27, 30, 32–36, 38), and 9 of them (11, 27, 30, 32–34, 36, 38) indicated that Yuen KY at the University of Hong Kong had the largest number of CoV-related publications. Collaboration between authors and highly cited authors were not fully analyzed in the included studies, and were only mentioned in 4 bibliometric analyses (30, 33, 35, 36) (Table 4, Appendix, Supplementary Table 5).

#### Research Topics of CoV-Related Publications in Published Bibliometric Analysis

Four included bibliometric analyses (9, 27, 30, 31) reported the total number of CoV-related research keywords (132–216). Most of the included bibliometric analyses showed that the main research fields of the CoV-related research focused on basic medical sciences (virology, microbiology, biochemistry & molecular biology, immunology, pharmacology, and pharmacy), clinical medicine (infectious diseases, pediatrics, and the respiratory system), veterinary sciences, and public health (public, environmental, and occupational health). The research hotpots mainly focused on the mechanisms, pathology, epidemiology, clinical diagnosis, and treatment of the coronavirus in MERS-CoV and SARS-Cov (Table 5, Appendix, Supplementary Table 6).

**Table 5:** The findings of the main research topics in the published bibliometric analyses.

**References**	**Subject**	**Main research topics**	**Conclusions**
(11)	Focus on virology; Public health; Drugs and other hotspot fields; Uncovers changes in the direction of coronavirus research.	1. Public health, preventive medicine and epidemiology; 2. Virus detection and clinical diagnosis; 3. Some immunological and pharmaceutical research.	NR
(33)	NR	1. Clinical research; 2. Pathogenesis research; 3. Virological research; 4. Treatment; 5. Origin and transmission research.	Notably, COVID-19 must become the research hotspot of coronavirus research, and clinical research on COVID-19 may be the key to defeating this epidemic.
(9)	The most frequently assigned research fields are virology (2140); Infectious diseases (899); Veterinary sciences (720); Microbiology (622); Immunology (558).	1. The molecular and biological topics; 2. outlines the articles dealing with the SARS epidemic; 3. Combines the articles dealing with the MERS epidemic; 4. Focuses on the spike protein that is characteristic of CoV, its pathogenesis, and its connection to the other clusters.	The results underline the need for sustainable and forward-looking approaches that should not end with the containment of COVID-19.
(36)	The top six research areas were virology (2957); Infectious diseases (1594); Immunology (1306); Microbiology (1182); Veterinary sciences (1163); Biochemistry & molecular biology (1004).	1. Virology (including molecular, biology, and immunology); 2. Infectious diseases (including medicine, medical, and clinical); 3. Veterinary medicine.	The international cooperation is an important way to accelerate research progress and achieve success. Developing corresponding vaccines and drugs are the current hotspots and research directions.
(37)	NR	1. The biological and virologic characteristics of coronavirus, including essential factors of infection and transmission routes during the outbreaks of SARS and MERS, as well as clinical features; 2. Some types of coronavirus spread among animals and humans; 3. Primary infection of coronavirus in mammals and birds is confined to the upper respiratory and gastrointestinal system; 4. The entrance into human body of SARS-CoV depends on the ACE2 receptor, while the spike protein functions as the adaptor; 5. The evolution based on the mutation of coronavirus RNA caused different symptoms to human kind.	More research on prevention and treatment is needed according to an analysis of term density.
(30)	Mainly involve basic medical sciences (virology, microbiology, biochemistry & molecular biology, immunology, pharmacology, & pharmacy); Clinical medicine (infectious diseases, pediatrics, respiratory system); Veterinary sciences; Public health (public, environmental, and occupational health).	1. Mainly about respiratory viruses, which illustrated viral respiratory infections from the angle of the clinic; 2. Mostly about the genetic aspects of various coronaviruses; 3. Mainly about SARS-CoV; 4. Mainly about immunity; 5. Mostly about MERS-CoV.	Bibliometric analysis of the literature shows the research on coronavirus boomed when a novel coronavirus triggered outbreaks in people. With the end of the epidemic, the research tended to be cooling. Virus identification, pathogenesis, and coronavirus-mediated diseases attracted much attention. We must continue studying the viruses after an outbreak ended.
(32)	Virology; Veterinary sciences; Infectious diseases.	1. “Pathological research;” 2. “Epidemiology research;” 3. “Clinical research;” 4. “Mechanism research.”	The outbreak of the epidemic could promote coronavirus research, meanwhile, coronavirus research contributes to overcoming the epidemic. Attention should be drawn to the latest popular research, including “Spike protein,” “Receptor binding domain,” and “Vaccine.” Therefore, more and more efforts will be put into mechanism research and vaccine research and development, which can be helpful to deal with the epidemic.
(27)	NR	1. The biological and virologic characteristics of coronavirus, including essential factors of infection and transmission routes during the outbreaks of SARS and MERS, as well as clinical features;	While in the 1980s, USA and developed countries from Europe were major source countries and the virus was identified only as an animal disease in the literature and its biological and genetic structure was investigated, in
		2. Some types of coronaviruses spread among animals and humans; 3. Primary infection of coronavirus in mammals and birds is confined to the upper respiratory and gastrointestinal system; 4. The entrance into human body of SARS-CoV depends on the ACE2 receptor, while the spike protein functions as the adaptor; 5. The evolution based on the mutation of coronavirus RNA caused different symptoms to human kind.	the 2000s, China became a major contributor of coronavirus literature because the SARS outbreak originated from southern China. Almost all most-cited publications in this period are related to SARS and the ACE2 protein.
(27)	NR	NR	NR
(27)	NR	1. Coronavirus; 2. Mouse hepatitis virus; 3. Transmissible gastroenteritis virus; 4. Rotavirus; 5. Cat.	NR
(27)	NR	1. “Coronavirus;” 2. “SARS;” 3. “SARS coronavirus;” 4. “SARSCoV.”	NR
(27)	NR	1. “Saudi Arabia;” 2. “MERS-CoV;” 3. “Outbreak;” 4. “Vaccine;” 5. “Camel;” 6. “Zoonosis.”	NR
(26)	Infectious diseases (5341; 83.14%); Microbiology (5034; 78.36%); Virology (4956; 77.14%); Biochemistry molecular biology (4195; 65.30%); Genetics heredity (3191; 49.67%) etc.	The most commonly used keywords were “Coronavirus” followed by “Virus,” “Sars,” and “Infection.”	The results of the study showed that the growth pattern was not uniform, USA, and the University of Hong Kong have played a major role in the contribution of Coronavirus research. Even though this depicts a higher scientific growth, it is an alarming sign to the community for preparedness. Under the prevailing situation of seeking better prevention, treatment and vaccination for COVID-19, in-depth research in the above portrayed metrics would be an added knowledge for the researchers.
(34)	NR	1. Virus and coronavirus complementary research; 2. Virus and coronavirus types and strains.	This research serves as a framework to strengthen existing research lines and develop new ones, establishing synergistic relationships that were not visible without the maps generated herein.
(31)	NR	NR	Independent of the outcome of the current COVID-19 outbreak, we believe that measures should be taken to encourage sustained research in the field.
(35)	The treatment hot spots focused on preventing virus adsorption, inhibiting the virus gene nucleic acid replication, transcription and translation.	1. CoVs epidemiology; 2. Basic research; 3. Drug development.	Through the visualization analysis of knowledge graph, the development trend and hot spots of CoVs therapy research could be well observed. In this study, the degree of attention in the field of CoVs treatment showed periodic changes, related to the outbreak of new CoVs, and the country, institutions and the author were closely related. The treatment hot spots focused on preventing virus adsorption, inhibiting the virus gene nucleic acid replication, transcription and translation in order to develop new targets of drug.
(38)	Virology (3205, 29.5); Infectious Diseases (1442, 13.3); Veterinary Science (1391, 12.8); Immunology (1280, 11.8); Biochemistry Molecular Biology (1270, 11.7) etc.	NR	Future studies need to include articles from other quality databases as well in order to achieve generalizations. Future researchers also need to focus their attention now on experimental studies on COV.

*COVID-19, Coronavirus disease 2019; NR, not reported; SARS, severe acute respiratory syndrome; MERS, Middle East Respiratory Syndrome; CoV, coronavirus; ACE2, angiotensin-converting enzyme 2*.

## Discussion

We found that CoV-related publications showed two steep upward trajectories in 2003–2004 and 2012–2014. The research hotpots mainly focused on the mechanisms, pathology, epidemiology, clinical diagnosis, and treatment of the coronavirus in MERS-CoV and SARS-Cov. The most contributions to CoV-related research were from the USA and China in terms of the country; the University of Hong Kong in terms of the institute; and Yuen KY from the University of Hong Kong, in terms of the author.

The outbreak of SARS and MERS had a vital impact on the number of CoV-related publications. This study and included bibliometric analyses indicated that the number of CoV-related publications showed two steep upward trajectories from 2003 to 2004 and from 2012 to 2014, separately. The trends were consistent with the outbreak of the life-threatening SARS and MERS. The first case of SARS was identified on November 16, 2002, in China (39). The MERS-CoV was first identified in Saudi Arabia in April 2012, and cases have been confirmed every year with some significant rises in 2014, 2015, and 2019 (40). Until 30 June 2019, the majority of cases (84%) had been reported in Saudi Arabia (41). Since they are the places where the virus first appeared, China (No. publications = 2,754) and Saudi Arabia (No. publications = 422) have extensively studied CoV, and their number of publications are ranked 2^nd^ and 11^th^ respectively.

Overall, the USA and China played an important role in CoV-related research, followed by the Netherlands and England. This study found the USA and China were the most contributing countries in terms of STC, h-index, and NCMCI, which was supported by a previous study (20). This study showed that some institutes in the USA, China, and the Netherlands formed close cooperative relationships with other institutes. Because the USA is leading global scientific production, and the effort of the USA to foster international cooperation on CoV-related disease.

Keywords cluster analyses showed that the main research fields of the CoV-related research focused on basic medical sciences (virology, microbiology, biochemistry & molecular biology, immunology, pharmacology, and pharmacy), clinical medicine (infectious diseases, pediatrics, and the respiratory system), veterinary sciences, and public health (public, environmental, and occupational health). The research hotpots mainly focused on the mechanism, pathology, epidemiology, clinical diagnosis, and treatment of the coronavirus in MERS-CoV and SARS-Cov. These findings were in line with the findings of other published bibliometric analyses included in this study. However, the complete research process of virus includes the following: (1) studying the structure and function of the virus genome to fully understand the general morphology and structural characteristics of the virus; (2) exploring the replication, gene expression, and regulatory mechanism of the virus genome, to reveal the molecular nature of the virus infection and disease-causing; (3) researching and developing the virus genetic engineering vaccine and antiviral drugs; (4) studying the diagnosis, prevention, and treatment scheme of the virus infection disease (42, 43). This study showed the current research on CoV mainly focused on the first two stages of virus research. Therefore, there still was a lack of enough research on the related clinical, epidemiological, diagnostic, and therapeutic aspects (44). As the WHO recommended, drugs and vaccines were considered to need accelerated research and development (45), and research on the diagnosis, vaccines, and treatment options for CoV-related diseases should be strengthened (46).

Yuen KY from the University of Hong Kong contributed most to Cov-related research, especially in the fields of virology and microbiology. Followed by Baric RS and Drosten C, both of whom were members of the CoV Study Group (CSG) and assessed the novelty of the human pathogen tentatively named SARS-CoV-2 (47). The research of the CSG will improve understanding of virus-host interactions in an ever-changing environment and enhance our preparedness for future outbreaks (47). In the future, CoV-related researchers can collaborate to conquer the virus.

## Strengths and Limitations

To our best knowledge, this is the first systematic review of bibliometric analysis in global coronavirus research trends before COVID-19. We also explored the top-5 research areas of the top-10 countries and top-10 authors in this bibliometric analysis.

However, our study has some limitations.Firstly, for the bibliometric analysis,we only searched WOS, which may lead to the omission of some important studies (48–50). Secondly, some of the data we analyzed were automatically extracted from the downloaded publications by the software, such as author names. Since the software could not distinguish between authors with the same name, this might affect the results of our analyses. Thirdly, for the systematic review, the assessment of the risk of bias for the included studies was important, but we did not conduct the risk of bias assessment for lack of a valid assessment tool.

## Conclusions

CoV-related publications before COVID-19 have shown a rapidly increasing trend. The USA and China have played a vital role in CoV-related researches. Yuen KY from the University of Hong Kong has made contributions. The research topics mainly involved the mechanisms, pathology, epidemiology, clinical diagnosis, and treatment of the coronavirus in MERS-CoV and SARS, and more researchers should focus on the prevention, diagnosis, and treatment in the future.

## Author Contributions

KY, PY, SW, and ML were responsible for the conception and design of the study. PY was in charge of the literature search data acquisition. PY, ML, ZL, JL, XH, YB, and YX collected, analyzed, and interpreted the data and wrote the first draft of the manuscript. YL was responsible for the editing and standardization of the tables and figures and gave critical advice on the manuscript. All authors reviewed the manuscript for important intellectual content and approved the final version for publication.

## Funding

This work was supported by the Key Project of the Social Science Fund of Gansu Province: Social Research on the Response to COVID-19 in Gansu Province (Grant No. 20ZD016) and Fundamental Research Funds for the Central Universities (Grant No. lzujbky-2020-sp14).

## Conflict of Interest

The authors declare that the research was conducted in the absence of any commercial or financial relationships that could be construed as a potential conflict of interest.

## Publisher's Note

All claims expressed in this article are solely those of the authors and do not necessarily represent those of their affiliated organizations, or those of the publisher, the editors and the reviewers. Any product that may be evaluated in this article, or claim that may be made by its manufacturer, is not guaranteed or endorsed by the publisher.
