# Supplementary material for: Bibliometric Analysis and Systematic Review of Global Coronavirus Research Trends Before COVID-19: Prospects and Implications for COVID-19 Research
Source: Front Med (Lausanne). 2021 Nov 16;8:729138. doi: 10.3389/fmed.2021.729138 (PMC8635101; doi:10.3389/fmed.2021.729138)
Supplement: Supplementary file 1 [file Data_Sheet_1.pdf]

Supplementary Information for

Bibliometric analysis and systematic review of global coronavirus research trends before COVID-19: prospects and implications for COVID-19 Research

Peijing Yan, Meixuan Li, Jing Li, Zhenxing Lu, Xu Hui, Yuping Bai, Yangqin Xun, Yongfeng Lao, Shizhong Wang, Kehu Yang

Corresponding Author:

Kehu Yang

E-mail: yangkh-ebm@lzu.edu.cn

Shizhong Wang

E-mail: Wangshizhong-wuwei2021@163.com

This PDF file includes:

Figs. S1 to S4

Table S1 to S6

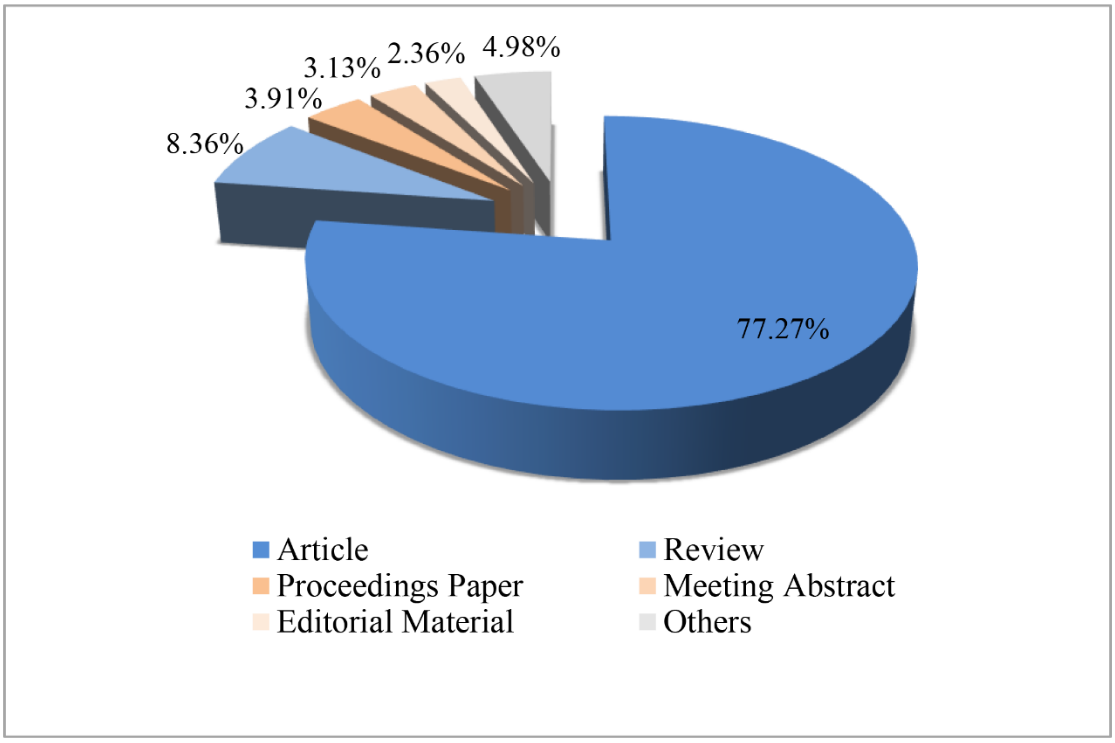

Fig. S1. Publication types of CoV-related publications.

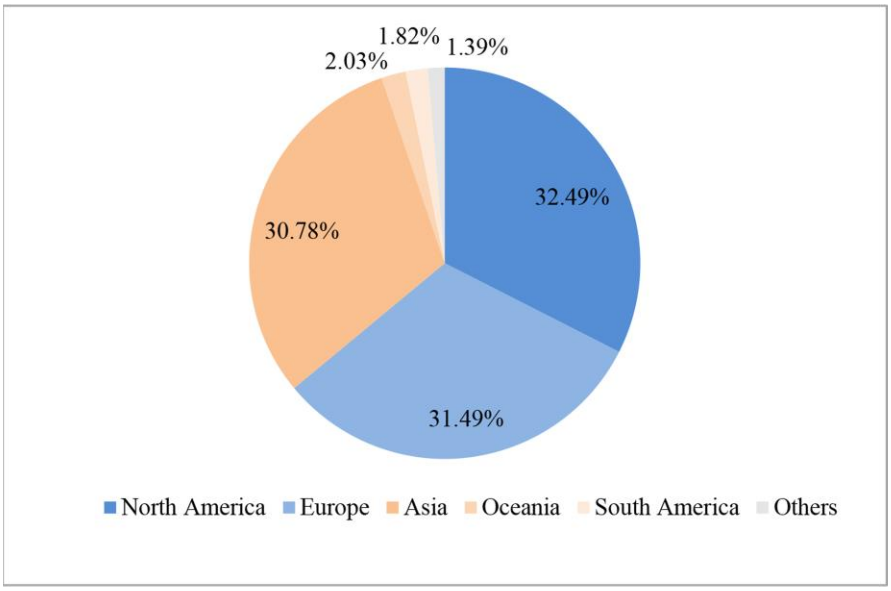

Fig. S2. Proportion of CoV-related publications in regions.

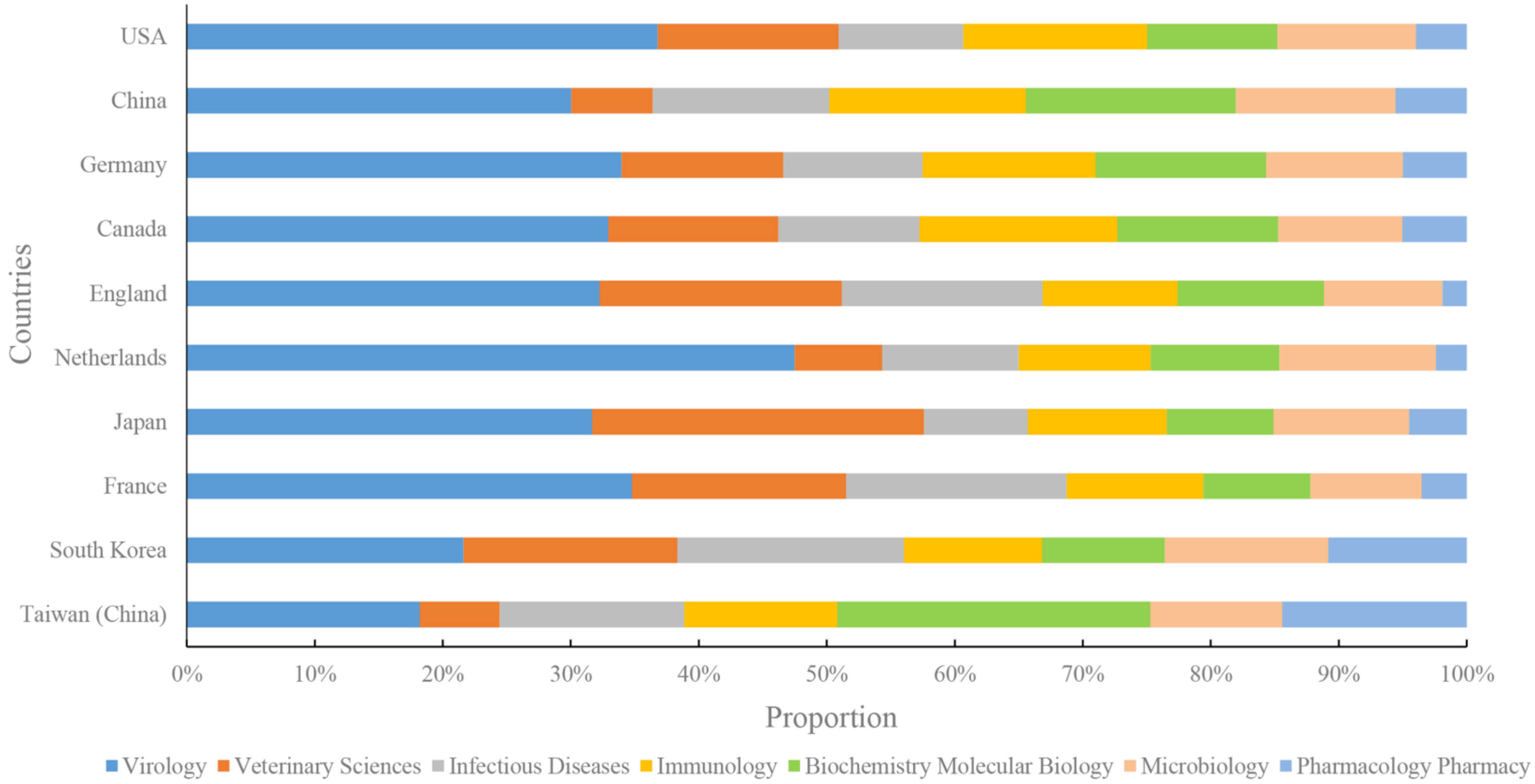

Fig. S3. Proportion of top-5 research areas in the top-10 countries.

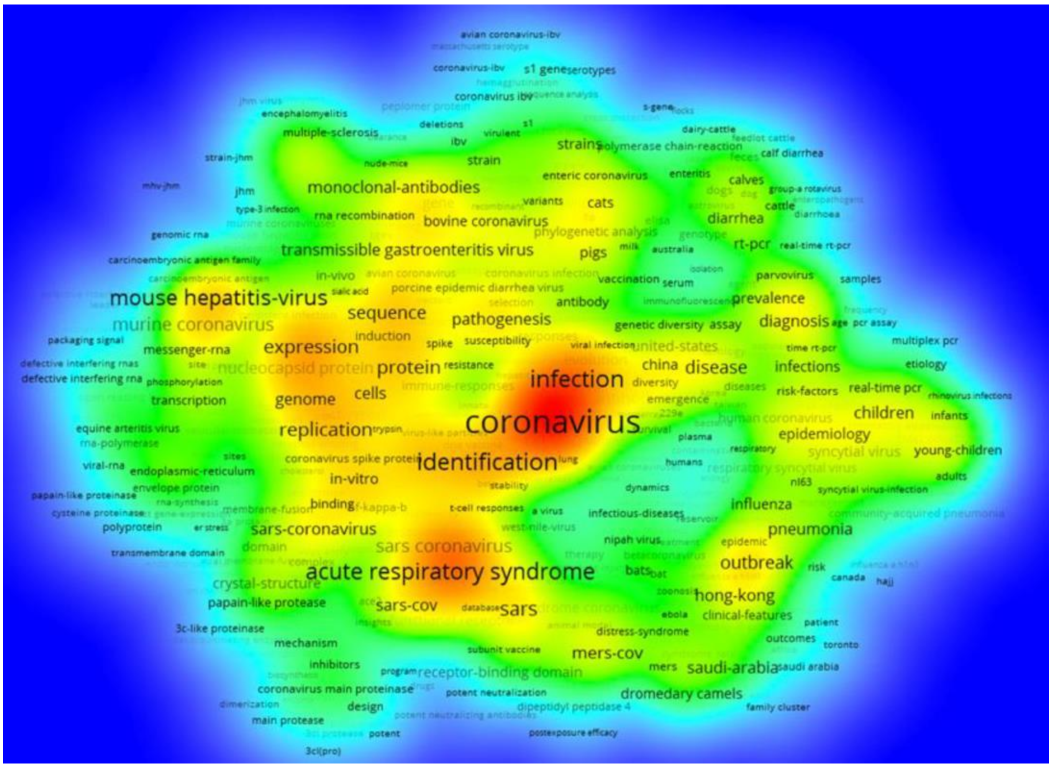

Fig. S4. Keywords density visualization map of CoV-related publications.

Table S1. Search strategy in Web of Science for bibliometric analysis

**#1**	TOPIC: (Coronavirus*) OR TOPIC: (Deltacoronavirus*) OR TOPIC: ("Munia coronavirus HKU13") OR TOPIC: ("Coronavirus HKU15") OR TOPIC: ("Rabbit Coronavirus*") OR TOPIC: ("Bulbul coronavirus HKU11") OR TOPIC: ("Thrush coronavirus HKU12")<br>Indexes=SCI-EXPANDED, SSCI, A&HCI, CPCI-S, CPCI-SSH, CCR-EXPANDED; IC Timespan=All years; DocType=All document types; Language=All languages;
**#2**	TS=("Bovine Coronavirus*") OR TS=("Canine respiratory coronavirus*") OR TS=("Canine Coronavirus*") OR TS=("Rat Coronavirus") OR TS=("Porcine Respiratory Coronavirus") OR TS=("Avian coronavirus*") OR TS=(Gammacoronavirus) OR TS=("Human Coronavirus OC43") OR TS=("Human Coronavirus 229E") OR TS=(Alphacoronavirus) OR TS=("Miniopterus bat coronavirus HKU8") OR TS=("Miniopterus bat coronavirus 1") OR TS=("Rhinolophus bat coronavirus HKU2") OR TS=("Mink coronavirus 1") OR TS=("Bat coronavirus HKU10") OR TS=("Bat coronavirus CDPHE15*") OR TS=("Scotophilus bat coronavirus 512") OR TS=("Feline Enteric Coronavirus*") OR TS=("Feline Coronavirus*") OR TS=("Human Coronavirus NL63") OR TS=("Betacoronavirus 1") OR TS=("Human enteric coronavirus*") OR TS=("Equine coronavirus*") OR TS=(Betacoronavirus) OR TS=("Human coronavirus HKU1")<br>Indexes=SCI-EXPANDED, SSCI, A&HCI, CPCI-S, CPCI-SSH, CCR-EXPANDED; IC Timespan=All years; DocType=All document types; Language=All languages;
**#3**	TOPIC: ("Rousettus bat coronavirus HKU9") OR TOPIC: ("Tylonycteris bat coronavirus HKU4") OR TOPIC: ("Pipistrellus bat coronavirus HKU5") OR TOPIC: ("SARS Virus") OR TOPIC: ("SARS Related Coronavirus*") OR TOPIC: ("SARS-Related Coronavirus*") OR TOPIC: ("Urbani SARS Associated Coronavirus*") OR TOPIC: ("SARS Associated Coronavirus*") OR TOPIC: ("Severe acute respiratory syndrome-related coronavirus") OR TOPIC: ("SARS-Associated Coronavirus") OR TOPIC: ("Severe acute respiratory syndrome related coronavirus") OR TOPIC: ("SARS Coronavirus*") OR TOPIC: ("Urbani SARS-Associated Coronavirus*") OR TOPIC: ("Turkey Coronavirus*") OR TOPIC: ("Middle East Respiratory Syndrome Coronavirus*") OR TOPIC: ("Middle East respiratory syndrome-related coronavirus*") OR TOPIC: ("Middle East respiratory syndrome related coronavirus*") OR TOPIC: ("Murine hepatitis virus*") OR TOPIC: ("Murine coronavirus*") OR TOPIC: ("Mouse Hepatitis Virus*") OR TOPIC: ("Murine Gastroenteritis Virus*") OR TOPIC: (MHV-JHM) OR TOPIC: ("Murine coronavirus*")<br>Indexes=SCI-EXPANDED, SSCI, A&HCI, CPCI-S, CPCI-SSH, CCR-EXPANDED; IC Timespan=All years; DocType=All document types; Language=All languages;
**#4**	#3 OR #2 OR #1<br>Indexes=SCI-EXPANDED, SSCI, A&HCI, CPCI-S, CPCI-SSH, CCR-EXPANDED; IC Timespan=All years; DocType=All document types; Language=All languages;

Table S2. Search strategy for systematic review

**Ovid MEDLINE and Ovid Embase**	#1 ("bibliometric analysis" or "analyses, bibliometric" or "analysis, bibliometric" or "bibliographies, statistical" or "bibliography, statistical" or "bibliometric analyses" or "bibliometric analysis" or "bibliometrics" or "statistical bibliographies" or "statistical bibliography").mp. or exp "bibliometrics"/ or exp "citation analysis"/ or exp "Bibliometrics"/ 22043<br>#2 "research trend".mp. or exp "trend study"/ use oemezd 35706<br>#3 1 or 257424<br>#4 ("Coronavirus" or "bulbul coronavirus hku11" or "coronavirus" or "coronavirus hku15" or "coronavirus, rabbit" or "coronaviruses" or "coronaviruses, rabbit" or "deltacoronavirus" or "deltacoronaviruses" or "munia coronavirus hku13" or "rabbit coronavirus" or "rabbit coronaviruses" or "thrush coronavirus hku12").mp. or exp "Coronavirinae"/ use oemezd or exp "Coronavirus"/ or exp "Coronavirus 229E, Human"/ or exp "Coronavirus, Bovine"/ or exp "Coronavirus Infections"/ or exp "Coronavirus NL63, Human"/ or exp "Coronavirus OC43, Human"/ or exp "Middle East Respiratory Syndrome Coronavirus"/ or exp "Spike Glycoprotein, Coronavirus"/ or exp "Coronavirus, Canine"/ or exp "Coronavirus, Feline"/ or exp "Coronavirus, Turkey"/ or exp "Porcine Respiratory Coronavirus"/ or exp "Coronavirus, Rat"/ 196044<br>Embase <1974 to 2021 January 21>(113295)<br>Ovid MEDLINE(R) ALL <1946 to January 22, 2021>(82749)<br>#5 3 and 4 419<br>#6 remove duplicates from 5 372
**Web of Science**	# 1 TOPIC: (Coronavirus*) OR TOPIC: (Deltacoronavirus*) OR TOPIC: ("Munia coronavirus HKU13") OR TOPIC: ("Coronavirus HKU15") OR TOPIC: ("Rabbit Coronavirus*") OR TOPIC: ("Bulbul coronavirus HKU11") OR TOPIC: ("Thrush coronavirus HKU12")<br># 2 TS=("Bovine Coronavirus*") OR TS=("Canine respiratory coronavirus*") OR TS=("Canine Coronavirus*") OR TS=("Rat Coronavirus") OR TS=("Porcine Respiratory Coronavirus") OR TS=("Avian coronavirus*") OR TS=(Gammacoronavirus) OR TS=("Human Coronavirus OC43") OR TS=("Human Coronavirus 229E") OR TS=(Alphacoronavirus) OR TS=("Miniopterus bat coronavirus HKU8") OR TS=("Miniopterus bat coronavirus 1") OR TS=("Rhinolophus bat coronavirus HKU2") OR TS=("Mink coronavirus 1") OR TS=("Bat coronavirus HKU10") OR TS=("Bat coronavirus CDPHE15*") OR TS=("Scotophilus bat coronavirus 512") OR TS=("Feline Enteric Coronavirus*") OR TS=("Feline Coronavirus*") OR TS=("Human Coronavirus NL63") OR TS=("Betacoronavirus 1") OR TS=("Human enteric coronavirus*") OR TS=("Equine coronavirus*") OR TS=(Betacoronavirus) OR TS=("Human coronavirus HKU1")<br># 3 TOPIC: ("Rousettus bat coronavirus HKU9") OR TOPIC: ("Tylonycteris bat coronavirus HKU4") OR TOPIC: ("Pipistrellus bat coronavirus HKU5") OR TOPIC: ("SARS Virus") OR TOPIC: ("SARS Related Coronavirus*") OR TOPIC: ("SARS-Related Coronavirus*") OR TOPIC: ("Urbani SARS Associated Coronavirus*") OR TOPIC: ("SARS Associated Coronavirus*") OR TOPIC: ("Severe acute respiratory syndrome-related coronavirus") OR TOPIC: ("SARS-Associated Coronavirus") OR TOPIC: ("Severe acute respiratory syndrome related coronavirus") OR TOPIC: ("SARS Coronavirus*") OR TOPIC: ("Urbani SARS-Associated Coronavirus*") OR TOPIC: ("Turkey Coronavirus*") OR TOPIC: ("Middle East Respiratory Syndrome Coronavirus*") OR TOPIC: ("Middle East respiratory syndrome-related coronavirus*") OR TOPIC: ("Middle East respiratory syndrome related coronavirus*") OR TOPIC: ("Murine hepatitis virus*") OR TOPIC: ("Murine coronavirus*") OR TOPIC: ("Mouse Hepatitis Virus*") OR TOPIC: ("Murine Gastroenteritis Virus*") OR TOPIC: (MHV-JHM) OR TOPIC: ("Murine coronavirus*")

	# 4 #3 OR #2 OR #1<br># 5 TOPIC: (Bibliometrics) OR TOPIC: (Bibliometric) OR TOPIC: ("bibliometric analyses") OR TOPIC: ("bibliometric analysis") OR TOPIC: ("statistical bibliographies") OR TOPIC: ("statistical bibliography") OR TOPIC: ("research trend") OR TOPIC: ("research trends")<br># 6 #5 AND #4<br>Indexes=SCI-EXPANDED, SSCI, A&HCI, CPCI-S, CPCI-SSH, CCR-EXPANDED, IC Timespan=All years

Table S3. The annual CoV-related publications of the top-10 countries

Country	Years																			
	1980	1981	1982	1983	1984	1985	1986	1987	1988	1989	1990	1991	1992	1993	1994	1995	1996	1997	1998	1999
USA	14	26	53	28	35	30	50	50	36	33	47	71	76	78	74	101	72	97	113	98
China	0	0	0	0	0	0	0	0	0	0	0	1	0	0	1	0	0	1	0	0
Germany	1	2	6	3	1	1	0	0	0	2	10	19	12	18	15	19	18	18	23	14
Canada	4	9	10	8	13	16	11	10	6	22	16	29	19	15	24	26	15	12	24	11
England	12	9	11	12	6	9	10	5	5	6	10	21	20	12	11	12	12	14	13	14
Netherlands	1	4	4	4	4	2	4	6	9	7	8	16	14	12	14	20	15	14	14	11
Japan	8	12	8	8	8	8	8	6	8	7	7	17	12	14	11	11	15	12	17	9
France	0	6	12	4	7	4	4	14	8	2	9	10	13	12	17	11	3	10	14	12
South Korea	0	0	0	0	0	0	0	0	0	0	0	0	0	0	0	0	0	1	1	2
Taiwan(China)	0	0	0	0	0	0	0	0	0	1	0	0	0	0	0	1	2	0	0	1

(continue) Table S3. The annual CoV-related publications of the top-10 countries

Country	Years																				
	2000	2001	2002	2003	2004	2005	2006	2007	2008	2009	2010	2011	2012	2013	2014	2015	2016	2017	2018	2019	2020
USA	81	131	91	119	253	218	267	205	220	169	158	145	168	217	263	275	277	233	218	243	9
China	0	2	1	144	280	264	169	151	114	116	112	98	109	133	133	169	193	184	173	205	1
Germany	9	20	16	30	51	51	57	38	42	33	30	27	29	46	65	47	42	54	50	40	2
Canada	10	16	9	26	66	58	52	47	29	30	18	19	18	20	20	23	30	32	28	36	0
England	6	19	6	20	46	41	52	31	22	26	16	25	29	40	65	38	41	45	38	49	1
Netherlands	13	7	9	25	24	39	55	32	35	35	32	22	31	36	47	31	31	38	28	33	2
Japan	11	18	7	7	33	29	33	34	33	22	27	24	23	31	28	37	32	29	26	20	0
France	8	5	9	17	18	21	29	20	15	17	20	18	23	33	45	30	51	23	32	40	1
South Korea	7	1	3	7	2	7	14	17	12	14	20	21	15	23	24	23	59	53	51	59	2
Taiwan(China)	0	0	0	8	68	62	37	24	25	25	23	13	16	22	25	13	17	17	12	9	1

Table S4. Basic information, search date of published bibliometric analysis and the number of related trends of CoV-related publications.

Basic information				Search data				Number of publications		
Title	Author, year	Journal	IF	Search inception	Search deadline	Dataset	Filter	No. publica tions	Type of study(%)	The trend of the No. publications
Bibliometric and visualization analysis of human coronaviruses: Prospects and implications for COVID-19 research	Deng Z, 2020	Frontiers in cellular and infection microbiolo gy	4.123	Database inception	15-Feb-20	Scopus	The language was restricted to English and Chinese; human coronavirus	15,20 7	Articles (60.4%), reviews (14.3%), other types (25.3%)	The annual publications count was naturally divided into four sections, due to three notable epidemic events in history. Before the outbreak of SARS, the annual publication amount remained low, reaching a maximum of 29 in 1998; The annual publication number rose suddenly to 1729 in 2003 and continued to rise the following year, when it reached; 1754 publications. For the next few years, the publication count declined gradually, reaching a low of 488 publications in 2011. This lasted for 4 years, during which time the annual number reached another peak, 838 publications in 2015, coinciding with the second outbreak of MERS in South Korea.
COVID-19 will stimulate a new coronavirus research breakthrough: A 20-year bibliometric analysis	Tao Z, 2020	Annals of translationa l medicine	3.297	200 0	9-Feb-20	WOSC C	The document type limited to original articles and reviews	9,760	Articles (89.5%), reviews (10.5%)	NR
Coronavirus: An insight into global research until outbreak of COVID-19 and its implications for the future	Klingel höfer D, 2020	Journal of global health	2.899	Database inception	18-Mar-20	WOSC C	The document type limited to original	6,905	Articles (100.0%)	The first article about CoV found in this study was published in 1970. From then on, articles were published every year, but the number remained in double digits until 2003. In this year, the number of articles increased phenomenally to 290 articles and then in 2004 to 679. The following year was characterized by a sharp decline in annual figures. The second increase in the development of publication numbers on CoV began in 2012 and had its maximum of 340 articles in 2016. Then, the number shrank again to 273 articles in 2018.
Research progress	Zhai F,	Internationa	2.894	1-Jan-03	10-Apr-	WOSC	The language	11,036	Articles	The growth of publications showed a rising trend from

of coronavirus based on bibliometric analysis	2020	l Journal of Environmental Research and Public Health			20	C	was restricted to English; the document type limited to article, letter, and review		(85.7%), reviews (10.6%), letters (3.7%)	2003–2004 and from 2012–2016. Two prominent peaks occurred in 2004 and 2016, corresponding to the outbreaks of SARS-Cov in 2003 and MERS-Cov in 2015, respectively.
Twenty-year span of global coronavirus research trends: A bibliometric analysis	Zhou Y, 2020	International Journal of Environmental Research and Public Health	2.894	1-Jan-00	17-Mar-20	WOSCC	The language was restricted to English; The document type limited to Article	9,105	NR	Among these articles, 611 of them were published in 2004, accounting for 6.8%, the highest percentage, while 136 were published in 2000, accounting for 1.5%, the lowest. Only 235 articles were published in the first two years. After SARS and MERS broke out, there were two obvious publication bursts two years later. There was a sharp increase two months after the outbreak of COVID-19 as well.
Analysis of knowledge bases and research hotspots of coronavirus from the perspective of mapping knowledge domain	Jia Q, 2020	Medicine	1.552	1-Jan-03	11-Feb-20	WOSCC	The document type limited to article	8,433	NR	In 2003, 270 articles were published. From 2004 to 2006, the number of publications per year increased to nearly 600. Then, there was a decline in the number of publications per year between 2007 and 2011. After that, the number increased again and gradually rose to the level of 530 to 580 papers per year.
The status and trends of coronavirus research: A global bibliometric and visualized analysis	Mao X, 2020	Medicine	1.552	1-Jan-03	1-Jan-20	WOSCC	The language was restricted to English; The document type limited to article and review	9,294	Articles and reviews (100.0%)	The publication volume of the 18 years has fluctuated, with obvious increase points in 2004 and 2012
Holistic analysis of coronavirus literature: A scientometric study of the global publications relevant to SARS-CoV-2 (COVID-19),	Şenel E, 2020a	Disaster medicine and public health preparedness	0.977	1-Jan-80	31-Dec-19	WOSCC	NR	13,833	Articles (80.5%), reviews (8.5%), proceeding papers (4.6%), editorials	The peak year for publication was 2016 with 837 papers and 106 articles have been produced in 2020 so far.

MERS-CoV (MERS) and SARS-CoV (SARS)									(2.7%), other types (9.1%)	
Holistic analysis of coronavirus literature: A scientometric study of the global publications relevant to SARS-CoV-2 (COVID-19), MERS-CoV (MERS) and SARS-CoV (SARS)	Şenel E, 2020b	Disaster medicine and public health preparedness	0.977	1-Jan-80	31-Dec-19	WOSCC	1980-1989	641	Articles (100.0%)	NR
Holistic analysis of coronavirus literature: A scientometric study of the global publications relevant to SARS-CoV-2 (COVID-19), MERS-CoV (MERS) and SARS-CoV (SARS)	Şenel E, 2020c	Disaster medicine and public health preparedness	0.977	1-Jan-80	31-Dec-19	WOSCC	1990-1999	1,674	NR	NR
Holistic analysis of coronavirus literature: A scientometric study of the global publications relevant to SARS-CoV-2 (COVID-19), MERS-CoV (MERS) and SARS-CoV (SARS)	Şenel E, 2020d	Disaster medicine and public health preparedness	0.977	1-Jan-80	31-Dec-19	WOSCC	2000-2009	4,810	Articles (82.7%)	NR
Holistic analysis of	Şenel	Disaster	0.977	1-Jan-80	31-Dec-	WOSC	2010-2019	6,601	Articles	NR

coronavirus literature: A scientometric study of the global publications relevant to SARS-CoV-2 (COVID-19), MERS-CoV (MERS) and SARS-CoV (SARS)	E, 2020e	medicine and public health preparedness			19	C			(80.7%)	
Coronavirus: Bibliometric analysis of scientific publications from 1968 to 2020	Joshua V, 2020	Medical journal of the Islamic Republic of Iran	0	1-Jan-68	7-Mar-20	WOS	NR	6,424	Articles (90.1%), reviews (7.4%), other types (2.6%)	The number of publications gradually increased from 1 in 1968 to 388 in 2005 during the SARS outbreak. The publications on coronavirus slowly declined and again increased between 2014 and 2016, marking the outbreak of the MERS. The number of publications in 2019 were 275.
Global trends in coronavirus research at the time of COVID-19: A general bibliometric approach and content analysis using SciMAT	Herrera Viedma E, 2020	Professional De La Information	0.748	1-Jan-70	18-Apr-20	WOS	1970-2020; The language was restricted to English; The publication types: articles, proceedings, and reviews.	12,571	NR	Since the appearance of the first publications related to coronavirus in 1970, three milestones in the development of such literature can be observed. The first milestone corresponds to the first 35 years (1970-2005), during which the rate of publications increased, reaching a total of 3187 (25.1% of the total). In this analysis, the second milestone corresponds to the drop from 2005 to 2011, during which only 3554 documents were published, representing 28.3% of the publications from 1970 to 2020. Finally, the last year (2020) represents the last milestone, during which 1009 publications (8.0% of the total) were produced in only four months and marking the historical peak in this research field.
Scientometric trends for coronaviruses and other emerging viral infections	Kagan D, 2020	GigaScience	5.993	1-Jan-02	NR	MAG; PubMed; SJR; Wikidata	NR	NR	NR	In recent years, there has been a surge in academic publications, yielding >1 million new papers related to medicine and biology each year. In contrast to the overall increase in the number of infectious disease papers, there has been a relative decline in the number of papers about the coronaviruses SARS and MERS.
Visualization analysis on	Yi W, 2020	Zhonghua wei zhong	NA	Database inception	15-Feb-20	WOSCC	NR	1,747	NR	In terms of literature volume, the literatures on CoVs therapy rose after 2003 and 2012, and the number of

treatment of coronavirus based on knowledge graph	bing ji jiu yi xue		published literatures had remained high since 2014.

COVID-19, Coronavirus disease 2019; CoV, coronavirus; SARS, severe acute respiratory syndrome; MERS, Middle East Respiratory Syndrome; WOSCC, The Web of Science Core Collection; WOS, Web of Science; MAG, Microsoft Academic Graph; SJR, Scientific Journal Rankings; NR, not reported; NA, not available.

Table S5. Journals, institutions and authors of CoV-related publications in published bibliometric analysis.

Characteristic	Deng Z, 2020	Tao Z, 2020	Klingelhöfer D, 2020	Zhai F, 2020	Zhou Y, 2020
**Journals**					
No. journal	3,443	1,323	NR	1,609	1,202
The list of the Top popular journals	Top 20	Top 10	NR	Top 20	Top 15
The Top-3 journal list	JVI;<br>EID;<br>Lancet	JVI;<br>Virology;<br>PLoS One	NR	JVI;<br>EID;<br>Virology;<br>PNAS	JVI;<br>Virology;<br>PLoS ONE
The Top-3 highest IF	NR	EID (7.185);<br>JVI (4.324);<br>Viruses-Basel (3.811)	NR	NEJM (70.67);<br>Lancet (59.102);<br>PNAS (9.58)	NR
**Countries or territories**					
NO. countries	NR	114	NR	129	123
Countries of the authors	NR	1	NR	NR	NR
Countries of the correspondence authors	NR	NR	NR	NR	NR
The list of the Top countries with the most documents	Top 20	Top 10	Top 5	Top 22	Top 15
NO. publications of Top-3 countries (n,%)	USA (4,225, 27.8%);<br>China(mainland) (2,720,17.9%);<br>China(HongKong) (1,411, 9.3%)	USA (3,452, 35.4%);<br>China (2,402, 24.6%);<br>Germany (642, 6.6%)	USA (2,293, 33.2%);<br>China (1,707, 24.7%);<br>Germany (505, 7.3%)	USA (3,606, 32.7%);<br>China (3,139, 28.4%);<br>Germany (669, 6.1%)	USA (3,101, 34.3%);<br>China (2,230, 24.7%);<br>Germany (584, 6.5%)
With the co-authorship analysis map/network	Y	Y	Y	Y	Y
Network	USA, China and UK are the three most active countries, and all have a high frequency of international cooperation.	The most frequent cooperation occurs in USA and China	The most productive bilateral cooperation in CoV research was between the USA and China with 290 cooperation articles, followed by USA/Canada (113), USA – UK (77) and USA - Netherlands	USA (95), Germany (81), England (81), France (73), and China (70) were the countries with the most partnerships in the word.	The internal links of the USA with other regions involved in 3,101 articles; internal links of China with other regions involved in 2,230 articles; internal links of Germany with

			(75). The non-US partnership with the highest publication volume was between the Netherlands and Germany (74).		other regions involved in 584 articles; internal links of Netherlands with other regions involved in 502 articles; internal links of England with other regions involved in 480 articles; internal links of Japan with other regions involved in 449 articles.
**Institutions**					
NO. institutions	NR	NR	NR	NR	NR
The list of the Top institutions with the most publications	Top 20	Top 10	Top 15	Top 20	Top 15
The Top-3 institutions (documents)	University of Hong kong , China (703);<br>Chinese University of Hong kong, China (499);<br>Chinese Academy of Sciences, China (407)	University of Hong Kong, China (959);<br>Chinese Academy of Sciences, China (469);<br>Chinese University of Hong Kong, China (411)	University Hong Kong, China (398);<br>Chinese University Hong Kong, China (217);<br>CDC, USA (155)	University of Hong Kong (595);<br>Chinese University of Hong Kong (311);<br>CDC (266)	University of Hong Kong, China (434);<br>Chinese Academy of Science, China (329);<br>University of California System, USA (246)
With the co-authorship analysis map/network	N	Y	N	Y	N
Cooperation between institutions (international cooperation)	NR	The cooperation between institutions is insufficient	NR	The Top three organizations ranked by collaboration are the University of Hong Kong, CDC, USA, and the National Institute of Allergy and Infectious Diseases (NIAID).	NR
**Authors**					
No. authors	NR	29,515	NR	NR	NR
The list of the Top authors with the most documents	Top 20	Top 10	NR	Top 10	NR

The Top-3 authors	1. Yuen KY,University of Hong kong, Chna (180);<br>2. Drosten C, Charité-Universitätsmedizin Berlin, Germany (128);<br>3. Peiris JSM, University of Hong kong (111)	1. Yuen KY, University of Hong Kong, China (200);<br>2. Baric RS, University of North Carolina, USA (134);<br>3.Perlman S, University of Iowa, USA (133).	NR	1. Yuen, KY, University of Hong Kong, China (214);<br>2. Drosten C, University of Bonn, Germany (142);<br>3. Baric RS., University of North Carolina, USA (131)	NR
With the co-authorship analysis map/network	N	Y	NR	Y	NR
Cooperation between authors	NR	NR	NR	NR	NR
Top co-cited authors	NR	Top 10	NR	Top 10	NR
The Top-3 co-cited authors list	NR	1. Peiris JSM (1,759);<br>2. Drosten C (1,751);<br>3. Ksiazek TG (1,431)	NR	1. Yuen, Kwok-yung at University of Hong Kong (China), (17,507 weight of citations);<br>2. Chan, Kwok-Hung, at University of Hong Kong (China), (11,760 weight of citations);<br>3. Drosten, Christian at University of Bonn (Germany) (11,069 weight of citations)	NR

Continued Table S5. Journals, countries or territories, institutions and authors of CoV-related publications in published bibliometric analysis.

Characteristic	Jia Q, 2020	Mao X, 2020	Şenel E, 2020a	Şenel E, 2020b	Şenel E, 2020c
**Journal**					
No. journal	NR	NR	NR	NR	NR
The list of the Top popular journals	NR	Top 20	Top 10	Top 1	Top 1
The Top-3 journal list	NR	JVI;<br>Virology;<br>Virus Research	JVI;<br>Virology;<br>ADV EXP MED BIOL	JGV	JVI
The Top-3 highest IF	NR	NR	NR	NR	NR

**Countries or territories**					
NO. countries	84	78	NR	NR	NR
Countries of the authors	NR	Y	Y	NR	NR
Countries of the correspondence authors	NR	NR	NR	NR	NR
The list of the Top countries with the most documents	Top 10	Top 20	Top 5	Top 5	Top 5
NO. publications of Top-3 countries (n,%)	USA (2,791, 33.1%); China (2,231, 26.5%); Germany (564, 6.7%)	USA (3,225, 34.7%); China (2,410, 25.9%); Germany (621, 6.7%)	USA (4,894, 35.4%); China (16.7%); Germany (6.7%)	USA (36.7%); Germany (13.4%); UK (12.2%)	USA (44.3%); Germany (9.0%); Canada (8.3%)
With the co-authorship analysis map/network	Y	Y	N	N	N
Network	NR	USA (TLS=2,045 times), China (TLS=1,154 times), Germany (TLS=832 times), England (TLS=762 times), and Netherlands (TLS=653 times)	NR	NR	NR
**Institutions**					
NO. institutions	333	147	NR	NR	NR
The list of the Top institutions with the most publications	Top 10	Top 20	Top 10	Top 1	Top 1
The Top-3 institutions (documents)	University of Hong Kong, China (399); Chinese Academy Sciences, China (298); CDC, USA (184)	University of Hong Kong, China (452); Chinese Academy of Sciences, China (323); CDC, USA (197)	University of Hong Kong, China (534); Chinese Academy of Sciences, China (396); Utrecht University, Netherlands (335)	University of Würzburg,Germany	University of Southern California, USA (96)
With the co-authorship analysis map/network	Y	Y	NR	NR	NR
Cooperation between institutions (international cooperation)	NR	University of Hong Kong (TLS=341 times), Chinese Academy of Sciences (TLS=288 times), Ministry of Health (TLS=284 times), Alfaisal University	NR	NR	NR

		(TLS=207 times), and Chinese Academy of Medical Sciences (TLS=203 times)			
**Authors**					
No. authors	591	121	NR	NR	NR
The list of the Top authors with the most documents	Top 10	NR	Top 10	Top 1	Top 1
The Top-3 authors	1. Yuen KY, University of Hong Kong, China (178);<br>2. Drosten C, University of Bonn, Germany (118);<br>3. Baric RS, University of North Carolina,USA (114)	1. Yuen KY, University of Hong Kong, China ;<br>2. Chan KH;<br>3. Woo PCY, University of Hong Kong, China	1. Yuen KY, University of Hong Kong, China (218);<br>2. Perlman S, University of Iowa, USA (189);<br>3. Enjuanes L, Autonomous University of Madrid, Spain (176)	Termeulen V	Lai MMC, University of Southern California, USA (70)
With the co-authorship analysis map/network	Y	N	N	N	N
Cooperation between authors	NR	NR	NR	NR	NR
Top co-cited authors	Top 10	NR	NR	NR	NR
The Top-3 co-cited authors list	Peiris JSM (1,519); Drosten C (1,516 cited author); Ksiazek TG (1,243)	NR	NR	NR	NR

Continued Table S5. Journals, institutions and authors of CoV-related publications in published bibliometric analysis.

Characteristic	Şenel E, 2020d	Şenel E, 2020e	Joshua V, 2020	Herrera-Viedma E, 2020	Kagan D, 2020	Yi W, 2020
**Journal**						
No. journal	NR	NR	100	NR	NR	NR
The list of the Top popular journals	Top 3	NR	Top 17	Top 7	NR	NR
The Top-3 journal list	JVI;<br>Virology;<br>ADV EXP MED BIOL	NR	JVI;<br>JGV;<br>Virology	JVI;<br>Virology;<br>ADV EXP MED BIOL	NR	NR
The Top-3 highest IF	NR	NR	PNAS (9.504);<br>EID (7.422);	JVI (4.324);<br>JGV (2.809);	NR	NR

			JID (5.19)	Plos One (2.776)		
**Countries or territories**						
NO. contries	NR	NR	NR	NR	NR	NR
Countries of the authors			Y	NR	NR	NR
Countries of the conrespondence authors				NR	NR	NR
The list of the Top countries with the most documents	Top 5	Top 5	Top 4	Top 11	NR	Top 5
NO. publications of Top-3 countries (n,%)	USA (1,679, 34.9%); China (1,202, 25.0%); Canada (324, 6.7%)	USA (2,218, 33.6%); China (1,479, 22.4%); Germany (436, 6.6%)	USA (2,345, 36.5%); China (1,067, 16.6%); Germany (480, 7.5%)	USA (4,513, 35.9%); China (2,746, 21.8%); UK (962, 7.7%)	NR	USA (613, 35.4%); China (582, 33.6%); Germany (122, 7.1%)
With the co-authorship analysis map/network	N	N	N	N	NR	Y
Network	NR	NR	NR	NR	NR	The cooperation among countries was close.
**Institutions**						
NO. institutations	NR	NR	NR	NR	NR	242
The list of the Top institutions with the most publications	Top 3	Top 4	Top 15	Top 10	NR	Top 5
The Top-3 institutions (documents)	University of Hong Kong, China (284); Chinese Academy of Sciences, China (221); Chinese University of Hong Kong, China (172)	University of Hong Kong, China (243); National Institutes of Health, USA (184); Chinese Academy of Sciences, China (170)	University of Hong Kong, China (506); University of North Carolina, USA (412); Chinese Academy of Sciences, China (371)	University of Hong Kong, China (487); Chinese Academy of Sciences, China (373); University of California System, USA (321)	NR	Chinese Academy of Sciences, China (82); University of Hong Kong, China (74); Chinese University of Hong Kong, China (58)
With the co-authorship analysis map/network	NR	NR	NR	N	NR	Y
Cooperation between institutions (international cooperation)	NR	NR	NR	NR	NR	The Chinese Academy of Sciences, the University of Hong Kong, the Chinese University of Hong Kong, and the National Institute of Allergy and Infectious Diseases are closely

						related.
**Authors**						
No. authors	NR	NR	NR	NR	NR	465
The list of the Top authors with the most documents	Top 1	Top 1	NR	Top 10	NR	Top 5
The Top-3 authors	Yuen KY, University of Hong Kong,China (110)	Drosten C, Charité – Universitätsmedizin, Germany (113)	NR	1. Yuen KY, University of HongKong, China (201);<br>2. Perlman S, University of Iowa, USA (169);<br>3. Baric; Enjuanes L, Autonomous University of Madrid, Spain (162)	NR	1. Baric RS, University of North Carolina (21);<br>2. Yuen KY, University of Hong Kong, China (17); Snijder EJ, Netherlands (17); Kuochen Chou, USA (17);<br>3. Jiang Shibo, China (16)
With the co-authorship analysis map/network	N	N	NR	NR	NR	Y
Cooperation between authors	NR	NR	NR	NR	NR	NR
Top co-cited authors	NR	NR	NR	Top 10	NR	NR
The Top-3 co-cited authors list	NR	NR	NR	Yuen (16,710);<br>Chan (11,651);<br>Drosten (10,692)	NR	NR

NR, not reported; JVI, Journal of Virology; EID, Emerging Infectious Diseases; PNAS, Proceedings of the National Academy of Sciences of the United States of America; NEJM, The New England Journal of Medicine; ADV EXP MED BIOL, Advances in Experimental Medicine and Biology; JGV, Journal of General Virology; EID, Emerging Infectious Diseases; JID, The Journal of Infectious Disease; CDC, Center for Disease Control and Prevention; Y, yes; N, no.

Table S6 Subjects/research topics and citations of CoV-related publications in published bibliometric analysis.

Characteristic	Deng Z, 2020	Tao Z, 2020	Klingelhöfer D, 2020	Zhai F, 2020
**Subjects/Research Topics**				
No. keywords	NR	216	150	NR
Subject	Focus on virology;<br>Public health;<br>Drugs and other hotspot fields;<br>Uncovers changes in the direction of coronavirus research	NR	The most frequently assigned research fields are virology (2,140);<br>Infectious diseases (899);<br>Veterinary sciences (720);<br>Microbiology (622);<br>Immunology (558).	The top six research areas were virology (2,957);<br>Infectious diseases (1,594);<br>Immunology (1,306);<br>Microbiology (1,182);<br>Veterinary sciences (1,163);<br>Biochemistry & molecular biology (1,004)
Number of research hotspot clusters	3	5	4	3
Main research Topics	1. Public health, preventive medicine and epidemiology;<br>2. Virus detection and clinical diagnosis;<br>3. Some immunological and pharmaceutical research	1. Clinical research;<br>2 Pathogenesis research;<br>3. Virological research;<br>4. Treatment;<br>5. Origin and transmission research	1. The molecular and biological topics; 2. outlines the articles dealing with the SARS epidemic;<br>3. Combines the articles dealing with the MERS epidemic;<br>4. Focuses on the spike protein that is characteristic of CoV, its pathogenesis, and its connection to the other clusters.	1. Virology (including molecular, biology, and immunology);<br>2. Infectious diseases (including medicine, medical, and clinical);<br>3. Veterinary medicine.
Insufficient research areas	Studies on tracing, evolution and animal carriers of human coronavirus; some fields remain unexplored or underexplored. For example, the relationship between human coronavirus and immune metabolism, the application of RNA-seq and single cell sequencing technology in coronavirus research, and the possibility of cocktail therapy in viral treatment have not been studied extensively.	NR	NR	NR
**Citations**				
Overall situation	We found that more than half of the publications, up to 9,383 (61.7%, 4,991 articles, 1,075 reviews), had	NR	NR	NR

	been cited no more than 10 times, including 3,336 that had gone uncited (21.9%, 1,373 articles, 308 reviews).			
The list of the Top most cited	Top 20 articles with the most citations	Top 10	Top 10	NR
The Top-3 high-cited papers list	1. 2,686, Global trends in emerging infectious diseases (2008, Nature);<br>2. 2,156, A novel coronavirus associated with severe acute respiratory syndrome (2003, NEJM);<br>3. 1,999, Identification of a novel coronavirus in patients with severe acute respiratory syndrome (2003, NEJM)	1. 1,827, A novel coronavirus associated with severe acute respiratory syndrome (2003, NEJM);<br>2. 1,734, Identification of a novel coronavirus in patients with severe acute respiratory syndrome (2003, NEJM);<br>3. 1,488, Characterization of a novel coronavirus associated with severe acute respiratory syndrome (2003, Science)	1. 1,841, A novel coronavirus associated with severe acute respiratory syndrome (2003, NEJM);<br>2. 1,752, Identification of a novel coronavirus in patients with severe acute respiratory syndrome (2003, NEJM);<br>3. 1,490, Characterization of a novel coronavirus associated with severe acute respiratory syndrome (2003, Science)	NR
Conclusions	NR	Notably, COVID-19 must become the research hotspot of coronavirus research, and clinical research on COVID-19 may be the key to defeating this epidemic.	The results underline the need for sustainable and forward-looking approaches that should not end with the containment of COVID-19.	The international cooperation is an important way to accelerate research progress and achieve success. Developing corresponding vaccines and drugs are the current hotspots and research directions.

Continued Table S6 Subjects/research topics and citations of CoV-related publications in published bibliometric analysis.

Characteristic	Zhou Y, 2020	Jia Q, 2020	Mao X, 2020	Şenel E, 2020a
**Subjects/Research Topics**				
No. keywords	NR	132	175	NR
Subject	NR	Mainly involve basic medical sciences (virology, microbiology, biochemistry & molecular biology, immunology, pharmacology, & pharmacy);<br>Clinical medicine (infectious diseases, pediatrics, respiratory system);<br>Veterinary sciences;<br>Public health (public, environmental, and occupational health).	Virology;<br>Veterinary sciences;<br>Infectious diseases	NR

Number of research hotspot clusters	5	5	4	NR
Main research Topics	1. The biological and virologic characteristics of coronavirus, including essential factors of infection and transmission routes during the outbreaks of SARS and MERS, as well as clinical features;<br>2. Some types of coronavirus spread among animals and humans;<br>3. Primary infection of coronavirus in mammals and birds is confined to the upper respiratory and gastrointestinal system;<br>4. The entrance into human body of SARS-CoV depends on the ACE2 receptor, while the spike protein functions as the adaptor;<br>5. The evolution based on the mutation of coronavirus RNA caused different symptoms to human kind.	1. Mainly about respiratory viruses, which illustrated viral respiratory infections from the angle of the clinic;<br>2. Mostly about the genetic aspects of various coronaviruses;<br>3. Mainly about SARS-CoV;<br>4. Mainly about immunity;<br>5. Mostly about MERS-CoV.	1. "Pathological research";<br>2. "Epidemiology research";<br>3. "Clinical research";<br>4. "Mechanism research."	1. The biological and virologic characteristics of coronavirus, including essential factors of infection and transmission routes during the outbreaks of SARS and MERS, as well as clinical features;<br>2. Some types of coronavirus spread among animals and humans;<br>3. Primary infection of coronavirus in mammals and birds is confined to the upper respiratory and gastrointestinal system;<br>4. The entrance into human body of SARS-CoV depends on the ACE2 receptor, while the spike protein functions as the adaptor;<br>5. The evolution based on the mutation of coronavirus RNA caused different symptoms to human kind.
Insufficient research areas	Studies on tracing, evolution and animal carriers of human coronavirus; some fields remain unexplored or underexplored. For example, the relationship between human coronavirus and immune metabolism, the application of RNA-seq and single cell sequencing technology in coronavirus research, and the possibility of cocktail therapy in viral treatment have not been studied extensively.	NR	NR	NR
**Citations**				
Overall situation	NR	NR	NR	NR
The list of the Top most cited	Top 10	Top 10	NR	Top 10/10 years

The Top-3 high-cited papers list	1. 1,839, A novel coronavirus associated with severe acute respiratory syndrome (2003, NEJM);<br>2. 1,748, Identification of a novel coronavirus in patients with severe acute respiratory syndrome (2003, NEJM);<br>3. 1,489, Characterization of a novel coronavirus associated with severe acute respiratory syndrome (2003, Science)	1. 1,021, A novel coronavirus associated with severe acute respiratory syndrome (2003, NEJM);<br>2. 955, Identification of a novel coronavirus in patients with severe acute respiratory syndrome (2003, NEJM);<br>3. 933, Characterization of a novel coronavirus associated with SARS (2003, Science)	NR	1. 1,829, A novel Coronavirus associated with severe acute respiratory syndrome (2003, NEJM);<br>2. 1,739, Identification of a Novel Coronavirus in patients with severe acute respiratory syndrome (2003, NEJM);<br>3. 1,488, Characterization of a novel Coronavirus associated with severe acute respiratory syndrome (2003, Science)
Conclusions	More research on prevention and treatment is needed according to an analysis of term density.	Bibliometric analysis of the literature shows the research on coronavirus boomed when a novel coronavirus triggered outbreaks in people. With the end of the epidemic, the research tended to be cooling. Virus identification, pathogenesis, and coronavirus-mediated diseases attracted much attention. We must continue studying the viruses after an outbreak ended.	The outbreak of the epidemic could promote coronavirus research, meanwhile, coronavirus research contributes to overcoming the epidemic. Attention should be drawn to the latest popular research, including "Spike protein," "Receptor binding domain," and "Vaccine." Therefore, more and more efforts will be put into mechanism research and vaccine research and development, which can be helpful to deal with the epidemic.	While in the 1980s, USA and developed countries from Europe were major source countries and the virus was identified only as an animal disease in the literature and its biological and genetic structure was investigated, in the 2000s, China became a major contributor of coronavirus literature because the SARS outbreak originated from southern China. Almost all most-cited publications in this period are related to SARS and the ACE2 protein.

Continued Table S6 Subjects/research topics and citations of CoV-related publications in published bibliometric analysis.

Characteristic	Şenel E, 2020b	Şenel E, 2020c	Şenel E, 2020d	Şenel E, 2020e
**Subject/Research Topics**				
No. keywords	NR	NR	NR	NR
Subject (n,%)	NR	NR	NR	NR
Number of research hotspot clusters	NR	NR	NR	NR
Main research Topics	NR	1. Coronavirus;<br>2. Mouse hepatitis virus;<br>3. Transmissible gastroenteritis virus;<br>4. Rotavirus;<br>5. Cat	1. "Coronavirus";<br>2. "SARS";<br>3. "SARS coronavirus";<br>4."SARSCoV"	1. "Saudi Arabia";<br>2. "MERS-CoV";<br>3. "Outbreak";<br>4. "Vaccine";<br>5. "Camel";<br>6. "Zoonosis"

Insufficient research areas	NR	NR	NR	NR
**Citations**				
Overall situation	Average citations per item were 33.83	Average citations per item were 37.90	Average citations per item were 39.38	Average citations per item were 16.58
The list of the Top most cited				
The Top-3 high-cited papers list	1. 466, Characterization of An Efficient Coronavirus Ribosomal Frameshifting Signal - Requirement for An RNA Pseudoknot (1989, Cell);<br>2. 393, Coronaviruses - Structure and Genome Expression (1988, JGV);<br>3. 303, The Biology and Pathogenesis of Coronaviruses (1982, Current Topics in Microbiology and Immunology)	1. 1,328, Community Study of Role of Viral-Infections in Exacerbations of Asthma In 9-11-Year-Old Children (1995, BMJ);<br>2. 1,005, Psychological Stress and Susceptibility to The Common Cold (1991, NEJM);<br>3. 796, Respiratory Viruses and Exacerbations of Asthma in Adults (1993, BMJ)	1. 1,829, A novel coronavirus associated with severe acute respiratory syndrome (2003, NEJM);<br>2. 1,739, Identification of a novel coronavirus in patients with severe acute respiratory syndrome (2003, NEJM);<br>3. 1,488, Characterization of a novel coronavirus associated with severe acute respiratory syndrome (2003, Science)	1. 1,295, Isolation of a Novel Coronavirus from a Man with Pneumonia in Saudi Arabia (2012, NEJM);<br>2. 544, Hospital Outbreak of MERS Coronavirus (2013, NEJM);<br>3. 544, Human infections with the emerging avian influenza A H7N9 virus from wet market poultry: clinical analysis and characterization of viral genome (2013, Lancet)
Conclusions	NR	NR	NR	NR

Continued Table S6 Subjects/research topics and citations of CoV-related publications in published bibliometric analysis.

Characteristic	Joshua V, 2020	Herrera-Viedma E, 2020	Kagan D, 2020	Yi W, 2020
**Subject/Research Topics**				
No. keywords	NR	NR	NR	NR
Subject (n,%)	Infectious diseases (5,341, 83.1%);<br>Microbiology (5,034, 78.4%);<br>Virology (4,956, 77.1%);<br>Biochemistry molecular biology (4,195, 65.3%);<br>Genetics heredity (3,191, 49.7%) etc.	NR	NR	The treatment hot spots focused on preventing virus adsorption, inhibiting the virus gene nucleic acid replication, transcription and translation.
Number of research hotspot clusters	NR	2	NR	3
Main research Topics	The most commonly used keywords were 'Coronavirus' followed by 'Virus', 'Sars', and 'Infection'.	1. Virus and coronavirus complementary research;<br>2. Virus and coronavirus types and strains.	NR	1. CoVs epidemiology;<br>2. Basic research;<br>3. Drug development
Insufficient research	NR	NR	NR	NR

areas				
**Citations**				
Overall situation	All the Top-cited articles were published after 2003 and that the papers published during 1968 and 2002 were not much cited.	Similarly, the distribution of citations shows a positive developmental trend during the period from 1970 to 2020, during which a total of 382,447 citations (including self-citations) were recorded. Finally, WoS indicates that the average number of citations per cited article is 40.08. Complementing these data, the first significant increase in citations appears at the beginning of this century, when the number of citations exhibited a higher growth rate. In subsequent years, the average of the citation distribution rose continuously.	NR	NR
The list of the Top most cited	Top 10	Top 192	NR	Top 15
The Top-3 high-cited papers list	1. 2,175, A novel Coronavirus associated with severe acute respiratory syndrome (2003, NEJM);<br>2. 2,012, Identification of a Novel Coronavirus in patients with severe acute respiratory syndrome (2003, NEJM);<br>3. 1,747, Characterization of a novel Coronavirus associated with severe acute respiratory syndrome (2003, Science)	1. 1,932, A novel coronavirus associated with severe acute respiratory syndrome (2003, NEJM);<br>2. 1,826, Identification of a novel coronavirus in patients with severe acute respiratory syndrome (2003, NEJM);<br>3. 1,510, Characterization of a novel coronavirus associated with severe acute respiratory syndrome (2003, Science)	NR	1. 180, Coronavirus main proteinase (3CLpro) structure: basis for design of anti-SARS drugs (2003, Science);<br>2. 159, Clinical progression and viral load in a community outbreak of coronavirus-associated SARS pneumonia: a prospective study (2003, Lancet);<br>3. 142, The crystal structures of severe acute respiratory syndrome virus main protease and its complex with an inhibitor (2003, PNAS)
Conclusions	The results of the study showed that the growth pattern was not uniform, USA, and the University of Hong Kong have played a major role in the contribution of Coronavirus research. Even though this depicts a higher scientific growth, it is an alarming sign to the community for preparedness. Under the prevailing situation of seeking better prevention, treatment and vaccination for COVID-19, in-depth research in the above portrayed metrics would be an added	This research serves as a framework to strengthen existing research lines and develop new ones, establishing synergistic relationships that were not visible without the maps generated herein.	Independent of the outcome of the current COVID-19 outbreak, we believe that measures should be taken to encourage sustained research in the field.	Through the visualization analysis of knowledge graph, the development trend and hot spots of CoVs therapy research could be well observed. In this study, the degree of attention in the field of CoVs treatment showed periodic changes, related to the outbreak of new CoVs, and the country, institutions and the author were closely related. The treatment hot spots focused on preventing virus adsorption, inhibiting the virus gene nucleic acid replication, transcription and translation in order to develop new targets of drug.

	knowledge for the researchers.				

MERS, Middle East Respiratory Syndrome; NEJM, The New England Journal of Medicine; SARS, severe acute respiratory syndrome; ACE2, angiotensin-converting enzyme 2; JGV, Journal of General Virology; BMJ, British Medical Journal; PNAS, Proceedings of the National Academy of Sciences of the United States of America; NR, not reported.
